# Aesthetics and Psychological Effects of Fractal Based Design

**DOI:** 10.3389/fpsyg.2021.699962

**Published:** 2021-08-17

**Authors:** Kelly E. Robles, Michelle Roberts, Catherine Viengkham, Julian H. Smith, Conor Rowland, Saba Moslehi, Sabrina Stadlober, Anastasija Lesjak, Martin Lesjak, Richard P. Taylor, Branka Spehar, Margaret E. Sereno

**Affiliations:** ^1^Integrative Perception Lab, Department of Psychology, University of Oregon, Eugene, OR, United States; ^2^Perception and Aesthetics Lab, School of Psychology, UNSW Sydney, Sydney, NSW, Australia; ^3^Material Science Institute, Department of Physics, University of Oregon, Eugene, OR, United States; ^4^13&9 Design, Graz, Austria

**Keywords:** fractal design, composite fractals, fractal dimension, preference, aesthetics

## Abstract

Highly prevalent in nature, fractal patterns possess self-similar components that repeat at varying size scales. The perceptual experience of human-made environments can be impacted with inclusion of these natural patterns. Previous work has demonstrated consistent trends in preference for and complexity estimates of fractal patterns. However, limited information has been gathered on the impact of other visual judgments. Here we examine the aesthetic and perceptual experience of fractal ‘global-forest’ designs already installed in humanmade spaces and demonstrate how fractal pattern components are associated with positive psychological experiences that can be utilized to promote occupant wellbeing. These designs are composite fractal patterns consisting of individual fractal ‘tree-seeds’ which combine to create a ‘global fractal forest.’ The local ‘tree-seed’ patterns, global configuration of tree-seed locations, and overall resulting ‘global-forest’ patterns have fractal qualities. These designs span multiple mediums yet are all intended to lower occupant stress without detracting from the function and overall design of the space. In this series of studies, we first establish divergent relationships between various visual attributes, with pattern complexity, preference, and engagement ratings increasing with fractal complexity compared to ratings of refreshment and relaxation which stay the same or decrease with complexity. Subsequently, we determine that the local constituent fractal (‘tree-seed’) patterns contribute to the perception of the overall fractal design, and address how to balance aesthetic and psychological effects (such as individual experiences of perceived engagement and relaxation) in fractal design installations. This set of studies demonstrates that fractal preference is driven by a balance between increased arousal (desire for engagement and complexity) and decreased tension (desire for relaxation or refreshment). Installations of these composite mid-high complexity ‘global-forest’ patterns consisting of ‘tree-seed’ components balance these contrasting needs, and can serve as a practical implementation of biophilic patterns in human-made environments to promote occupant wellbeing.

## Introduction

Driving nature’s aesthetics, fractal patterns are prevalent across both microscopic and global structures in natural environments (Mandelbrot, 1982; Taylor, 2021). Fractals are comprised of self-similar patterns repeating across scale, with varying levels of recursion (number of repetitions across scales) and fractal dimension “*D*-value” (rate of pattern shrinkage between repetitions) that drive perceptions of pattern complexity by determining the relative contributions of coarse-to-fine structure for the overall pattern. Additionally, the nature of pattern repetition (occurring in either an exact or statistical manner) also impacts perceptions of pattern preference and complexity (Taylor et al., 2005, 2011; Taylor and Sprott, 2008; Hagerhall et al., 2015; Bies et al., 2016). The aesthetic quality of fractal patterns has been well observed (Spehar et al., 2003) and can be highlighted by its appearance in art (Taylor et al., 1999, 2018; Graham and Field, 2008; Graham and Redies, 2010; Viengkham and Spehar, 2018). Across diverse cultures, fractal patterns are present in both contemporary and traditional artworks. Exemplified by the fractal structure created by the layering of paint in paintings of Jackson Pollock (Taylor et al., 1999, 2007; Taylor, 2003), fractal patterns can elicit highly aesthetic responses through changes in complexity.

Furthermore, fractal patterns have the prospect of altering more than just the aesthetic experience of a given object (Juliani et al., 2016; Taylor et al., 2018; Abboushi et al., 2019; Roe et al., 2020; Spehar and Stevanov, 2021). Fractals can be installed into larger Euclidean spaces to mitigate the effect of unnatural spatial frequency content that can lead to visual strain and discomfort (O’Hare and Hibbard, 2011; Ogawa and Motoyoshi, 2020). The increasing amount of time people spend indoors surrounded by Euclidean architecture produces visual strain because of the additional visual effort required to process more artificial spatial frequencies is suggested to lead to detrimental effects such as increased rates of headaches (Penacchio and Wilkins, 2015). Beyond alleviating physical discomfort, occupant stress levels can be minimized through fractal installations reminiscent of nature by reducing cognitive and visual strain produced by surrounding unnatural spatial frequencies (Taylor, 2006; Hagerhall et al., 2008; Le et al., 2017). These positive impacts of viewing fractals can be considered within the context of biophilia (Wilson, 1984) which recognizes the inherent need of humans to connect to nature. In particular, it is possible that the stress-reduction (Ulrich, 1981; Ulrich et al., 1991; Kellert, 1993) and attention restoration (Kaplan and Kaplan, 1982, 1989) impacts studied in pioneering investigations of viewing nature might be induced through nature’s fractals by easing visual processing.

To utilize the beneficial effects of natural geometry, the *ScienceDesignLab* (*SDL*) was formed in 2017 to generate patterns informed by the psychology of aesthetics (Smith et al., 2020). To transform the patterns into the built environment, SDL collaborated with the *Mohawk Group* - one of the world’s largest flooring manufacturers. Floors represent a common, expansive space for exposing people to aesthetic patterns. Known as *Relaxing Floors*, the designs were launched in Spring 2019 and have since received ten awards for human-focused design. The designs were composed from fractal patterns based on the hypothesis that fractals are responsible for the positive impacts of viewing nature’s scenery.

**FIGURE 1:**
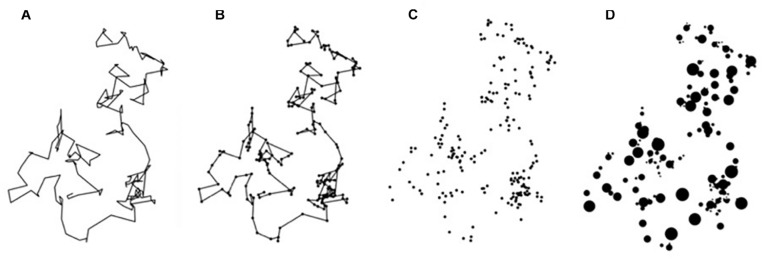
Fractal flights. **(A)** Lévy flight trajectories; **(B)** circular seed patterns are added to the ‘landing’ locations between these trajectories; **(C)** the trajectories are removed; **(D)** the sizes of the circles are scaled based on the length of the previous flights.

Whereas most studies of nature’s statistical fractals focus on images of individual objects, typical scenes feature ‘fractal composites’ in which individual objects merge to form an overall pattern. In addition to more closely capturing the essence of nature, *Relaxing Floors* exploited the extra flexibility offered by the composition process to develop patterns that were intriguing from a design perspective. To describe the compositional principle underpinning these fractals, we considered the analogy of individual fractal trees combining to create a fractal forest. Fractal trajectories called ‘Lévy flights,’ featuring flights with multiple length scales, were used as the starting point for these designs (Scott, 2005; Ferreira et al., 2012; Figure 1A). Much like a bird dropping a seed whenever it lands, the seeds then grow into fractal trees at the locations between the flight trajectories. For simplicity, the seeds shown in Figures 1B–D have a circular shape. The seed’s size can be scaled relative to the length of the previous flight, thus transferring the flight trajectory’s scaling properties to the dropped seed (Figure 1D).

**FIGURE 2:**
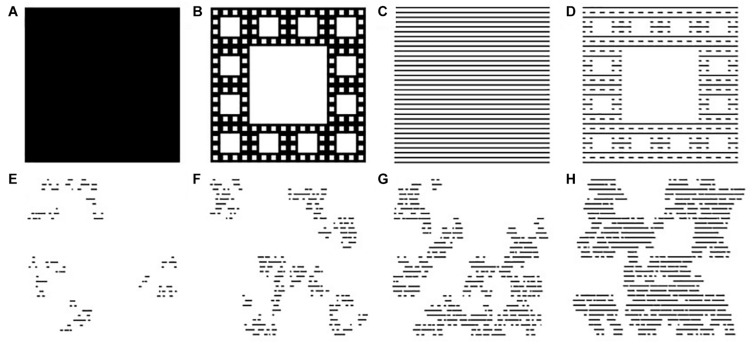
Fractal ‘trees.’ **(A)** The seed growth starts with a filled square; **(B)** a square-shaped seed is used to grow a Sierpinski fractal pattern with *D* = 1.8; **(C)** the black background is replaced with a line construction; **(D)** a square-shaped seed is used to grow a Sierpinski pattern superimposed on this lined background. The patterns are then randomized to morph the exact fractal into a statistical fractal. The *D*-value of the final fractal is inputted during this growth process. Four examples are shown here: **(E)** 1.2, **(F)** 1.4, **(G)** 1.6, and **(H)** 1.8.

**FIGURE 3:**
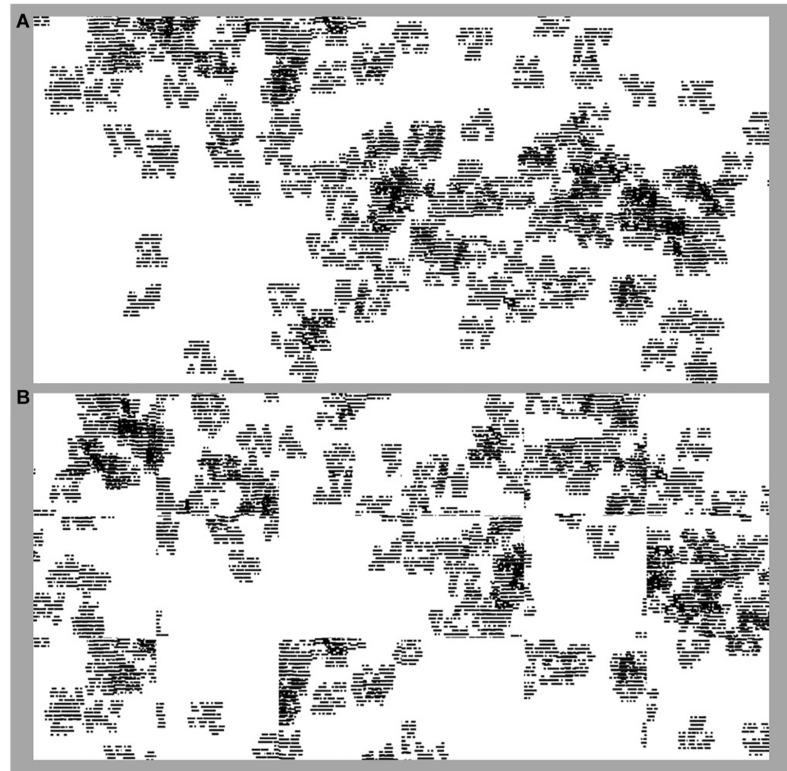
Fractal ‘forests.’ The forests integrate the flights of Figure 1 with the seed design of Figure 2. **(A)** is an image of the original (i.e., before randomization) forest pattern with *D* = 1.6; **(B)** shows the same forest after it has been divided into tiles and the tiles randomized.

Figure 2 shows the seed growth process that replaces each circle in Figure 1 with a ‘tree’ pattern based on a traditional fractal called the Sierpinski Carpet. This fractal grows from a square-shaped seed by repeating the square at multiple size scales (note that while Figure 2B shows three levels of repetition for demonstration purposes, the patterns used in the carpets typically feature 2 levels). In principle, the square-shaped seeds can be replaced with any shape, providing designers with considerable flexibility for future designs. Similarly, the black background can be replaced by various pattern textures including the lines used in the design that we will study here (Figure 2D). To convert the design from an exact to statistical pattern, randomness is introduced into the lengths of the black lines and also in the positions of the white squares (Figures 2E–H). The rate at which the seed changes size between the repetition levels can then be adjusted using *D*-value (Methods) – Figures 2E–G shows examples of fast (Figure 2E) to slow (Figure 2H) rates, each with a different randomization. The resulting fractal trees are then embedded at the landing sites between the fractal flights (Figure 3A). This design strategy therefore has the potential to incorporate fractal scaling in three key ways: (1) the fractal spacing between the trees (determined by the flights), (2) the distributions of the tree sizes (again set by the flights) and (3) the fractal growth of the seeds into trees.

A second motivation for the ‘bird flight’ composition strategy is that when viewing fractal patterns eye movements have been found to follow fractal trajectories (Taylor et al., 2011). This is because if the eye’s gaze is directed at just one location within the fractal scenery the peripheral vision only has sufficient resolution to detect coarse patterns. Therefore, the gaze shifts position to allow the eye’s fovea to detect the fine scale patterns at multiple locations. This allows the eye to experience the coarse and fine scale patterns necessary for confirmation of the fractal character of the stimulus. The reason the eye adopts a fractal trajectory when performing this task can be found in studies of animals such as birds foraging for food in their natural terrains. Their foraging motions are also fractal. For example, the short trajectories allow a bird to look for food in a small region and then to fly to neighboring regions and then onto regions even further away, allowing efficient searches across multiple size scales. The eye adopts the same motion when ‘foraging’ for visual information. These designs therefore place the tree locations using the same fractal statistics that the eye adopts when viewing them.

**FIGURE 4:**
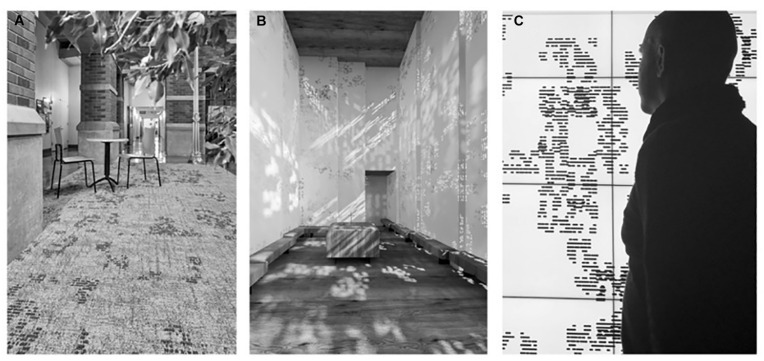
Installations. The fractal pattern of Figure 3 employed as a floor design at the University of Oregon, United States **(A)**, as wall patterns in the Fractal Chapel in the State Hospital in Graz, Austria **(B)**, and as a design for computer screen-savers **(C)**.

One challenge remained. For manufacturing demands, the 6ft (15 cm) by 12ft (30 cm) pattern of Figure 3A is divided into either 2ft by 2ft ‘tiles’ or 1ft by 3ft ‘planks,’ which will then be randomly re-assembled when installed in order accommodate the unique layout of any given space without altering the fractal *D*-value of the installation. We therefore had to simulate this division process to ensure that it did not disrupt the design aesthetic (in particular, that any discontinuities at the tile or plank edges fit well within the overall pattern) nor the fractal aesthetic (that the discontinuities did not alter the forest fractal’s *D* value). Figure 3B shows an example of the randomized flooring pattern. Figure 4 (left image) shows the patterns as they appear on the carpets. In addition to this tuning of pattern characteristics to achieve the fractal aesthetics, the patterns also need to translate well to the carpet format seen in Figure 4A. The tufted carpet background had to be textural enough to hide the tile edges without creating a pattern that would alter the intended *D*-value. New tufting techniques which hide unused yarns to create controlled texture were used to achieve an optimized construction for aesthetics. The *Relaxing Floors* collection featured three fractal forests generated using the above principles, each with an overall *D* value of 1.6. The three designs (Smith et al., 2020) differed in the number of repeating levels within the tree, the shapes chosen to build the tree, and also the extent to which the tree size was set relative to the flight trajectory. Here we focus on the design which used the trees shown in Figure 2 and which set all the trees to be the same size (irrespective of flight size).

Previous research demonstrates that visual complexity is a key component in the visual impact of fractals. Compared to the simplicity of Euclidean shapes, the fractal repetition of patterns at different scales results in fractal shapes that are inherently complex. The current series of studies expands upon typical measurement of fractal preference or complexity to address broader perceptual judgments (including ratings of complexity, engagement, preference, refreshment, and relaxation) of these “global forest” patterns and their respective local “tree-seed” patterns that are currently installed in multiple settings with the potential to promote viewer wellbeing (see Figure 4 for example installations). Figure 4 highlights an important key to success – the development of versatile designs that form the basis of multiple applications, in this case as carpet patterns for a university environment (in the *Mohawk* collaboration), as wall patterns used to disperse light throughout a chapel (in a collaboration with *INNOCAD Architecture*), and as computer screen savers (the latter are being made available for free personal use). The choice to use fractal patterns generated with design elements in mind, as opposed to images directly recruited from nature, serves to provide greater versatility in pattern design and application such that the base natural fractal pattern can be repeatedly varied to accommodate changing space requirements as well as adapting varying aesthetic design elements.

We will investigate these varied responses to global forest patterns of differing complexity by conducting studies in two laboratories (one at the University of Oregon in the United States [Experiment 1A] and the other at the University of New South Wales in Australia [Experiment 1B]) using slightly different rating scales as a test of the robustness of these effects. The use of both unipolar and bipolar rating scales is employed to ensure that our measurements are both sensitive enough to detect differences in psychological effects related to the fractal design patterns and generalizable across different measurement conditions. It is hypothesized that both of our rating scales will be able to identify consistent variations in the psychological effects of the various fractal design patterns, thus providing evidence of robust response patterns across measurement types. The goal of these studies is to establish an empirical basis for the optimal selection of fractal designs to meet varying psychological and aesthetic needs of a space (see Roe et al., 2020, for another example of this approach) by explicitly identifying whether general fractal preferences extend to more complex man-made fractal patterns and additional dimensions of psychological judgments. Finally, our results from subgroup analyses will guide the selection of specific fractal designs that balance various pattern factors (including *D*-value and arrangement) in order to benefit the most occupants possible without negatively impacting subgroups.

## Experiment 1 – Perception of Fractal “Global Forest” Patterns

We first examined the role of physical complexity and pattern arrangement in determining perceived complexity, engagement, preference, refreshment, and relaxation in ‘global forest’ fractal patterns. Experiment 1A used a series of unipolar slider tasks while Experiment 1B used a series of bipolar slider tasks.

### Experiment 1A–Perception of Fractal “Global Forest” Patterns With Unipolar Ratings

#### Materials and Methods

##### Stimuli

We used the pattern’s fractal dimension *D* to quantify visual intricacy. For the tree-seed patterns, the *D*-value dictates the rate of shrinkage of the patterns between repetition levels (Figures 2E–H). Similarly, the fractal flights follow a power law distribution with an exponent related to *D* that adjusts the relative sizes of the flights. In each case, high *D* results in a slower rate of shrinkage between the coarse and fine features. Lying on a scale between 1 and 2, higher *D*-value patterns feature larger contributions of fine scale patterns and thus appear to be rich in intricate detail. The *D*-values of the fractal forests were set by inputting the appropriate scaling parameters when generating the fractal trajectories and tree-seeds, and then a box-counting technique (Fairbanks and Taylor, 2011) was used to analyze the completed forest pattern to confirm that it scales according to the target *D*-value. This technique covers the pattern with a mesh of boxes and counts the boxes that are occupied by the pattern. By repeating the count for different box sizes, the pattern characteristics can be assessed at multiple size scales and confirmed to be scale invariant.

Fractal scaling was confirmed from the minimum pattern size of 0.2 inches (0.5 cm) up to 24 inches (61 cm). The box-counting method cannot confirm fractal scaling at scales larger than 24 inches due to a limited number of boxes at these scales (Fairbanks and Taylor, 2011). However, based on the fractal input parameters, it is expected that fractal scaling continues beyond the confirmed range. We note that even this restricted range of confirmed fractal scaling exceeds the magnification factor for typical physical fractals, for which the coarsest pattern is 25 times larger than the smallest (Avnir et al., 1998). Crucially, this factor of 25 was used for the stimuli used in most of the previous research that revealed the positive observation effects (Taylor et al., 2017, 2018). The scaling ranges of our designs therefore exceed those known to induce the positive effects.

**FIGURE 5:**
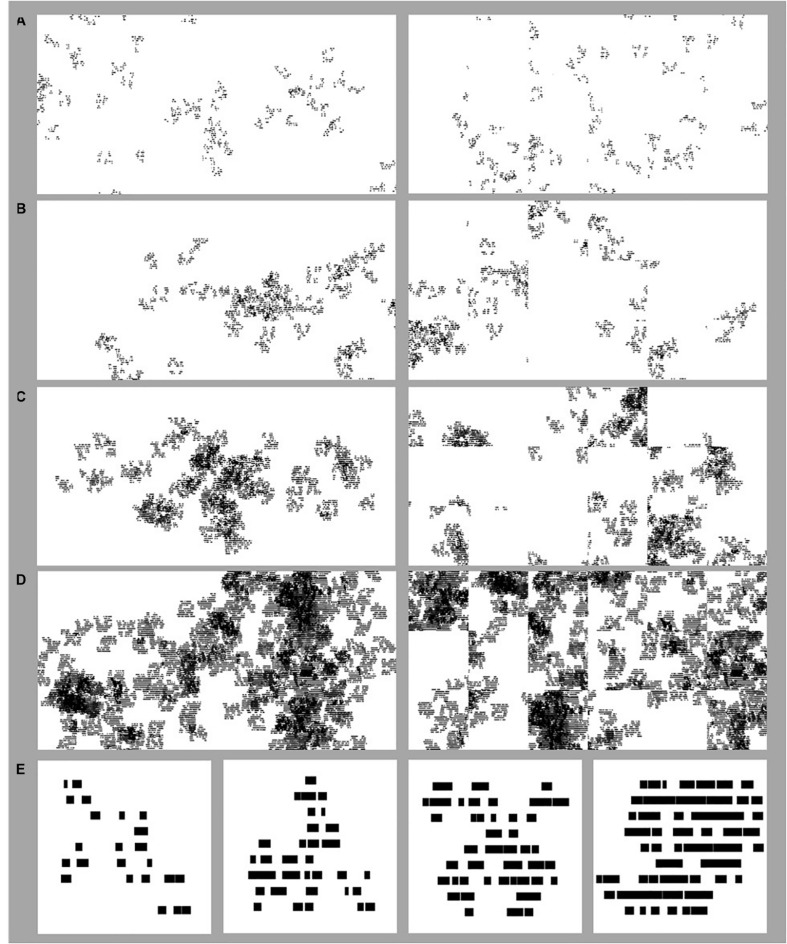
Example stimuli used in the Experiments. Fractal ‘forest’ stimuli used in Experiment 1 of differing *D*-values where *D* = 1.2 **(A)**, 1.4 **(B)**, 1.6 **(C)**, and 1.8 **(D)**. On the left side of each panel in **(A–D)** are images of the original forest pattern. On the right side of **(A–D)** are images of randomized versions of the original forest patterns, where the same forest has been divided into tiles and the tiles randomized. Fractal ‘tree’ stimuli used in Experiment 2 **(E)** of *D*-values of 1.2, 1.4, 1.6, and 1.8, from the left to right side of the panel.

Figures 5A–D shows examples of the ‘forest’ stimuli used in Experiment 1 with *D*-values of 1.2 (A), 1.4 (B), 1.6 (C), and 1.8 (D). The left side of each panel shows the original patterns while the right side shows the randomized version simulating the random pattern of ‘carpet squares’ installation in a space. Figure 5E shows example tree-seed stimuli of different *D*-values (1.2, 1.4, 1.6, and 1.8) that appear within the global forest stimuli.

##### Participants

To address how the addition of global fractal order may impact the perceptual judgments of fractal patterns, 78 participants comprised of undergraduate Psychology students from the University of Oregon were recruited for the current study through the SONA participant pool system (66 females, age ranging between 18 and 30 years old, mean age 20 years old). Informed consent was acquired following a protocol approved by the Institutional Review Board at the University of Oregon and all participants received class credit for their participation.

##### Visual displays

This study was generated in PsychoPy3 (Peirce et al., 2019) and used the online research study platform of Pavlovia and was completed on participants’ personal computers with program stimuli scaled to the individual computer’s respective full-screen dimensions.

##### Design and procedure

Participants viewed the “global forest” fractal patterns presented in five randomized blocks, with each block consisting of a singular judgment type (complexity, engaging, preference, refreshing, or relaxing). Each block’s stimulus set consisted of 5 unique patterns ranging across 4 levels of complexity or *D*-value (1.2, 1.4, 1.6, and 1.8) and varying in arrangement (non-randomized or randomized) giving rise to 40 trials per block and 200 total stimulus-related trials across the experiment. A slider response task was used to self-report ratings for each fractal pattern. Before each block, participants were instructed to make a single randomly ordered judgment (complexity, preference, engaging, refreshing, or relaxing) for each stimulus presented in that block. Specifically, they were asked to answer one of 5 questions for each block: “How _______ is the image?” with one of 5 different words placed in the blank (complex, engaging, preferable, refreshing, relaxing). They were told to indicate their rating of each given pattern on a slider ranging between 0 and 1 located below the image, with the “0” end of the slider indicating “not at all” and the “1” end of the slider indicating “completely.” They were asked to use the full range of the slider and to click on the slider to indicate their rating. Periodically, an attention check trial appeared in which participants were instructed to select either “0” or “1.” The images remained on the screen until participants selected their rating. Upon completion of the experiment, participants completed a demographic questionnaire and were debriefed according to the protocol approved by the Institutional Review Board at the University of Oregon.

#### Results

Data from 78 adult participants (between 18 and 33 years old) were retained from the 130 adults who participated in the experiment. Data were excluded due to: (a) failure to complete the study (6 participants), (b) failure of greater than 3 attention checks (24 participants), or (c) recording the same rating for greater than four consecutive trials. If the same rating was recorded for more than 4 consecutive trials, the entire block of ratings was excluded. Furthermore, if all blocks for a given judgment type were removed, then the participant was excluded (22 participants).

##### Fractal judgment task

**FIGURE 6:**
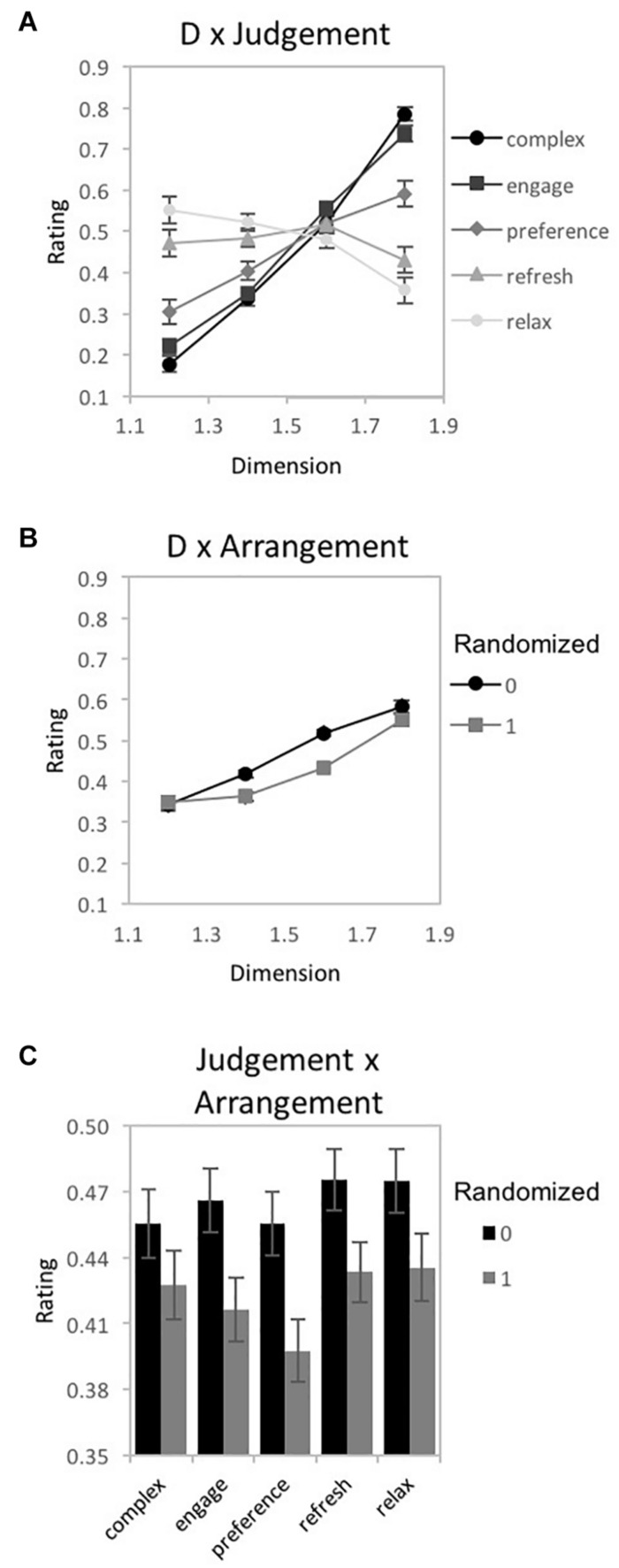
Experiment 1A results for “global forest” fractal patterns using a unipolar rating scale. Results show significant 2-way interactions among the experiment’s 3 factors: fractal dimension (*D*), stimulus pattern arrangement (“0” for non-randomized and “1” for randomized), and judgment type (complex, engaging, preferred, refreshing, and relaxing). Participant rating (on a scale from 0 to 1) is plotted as a function of **(A)** *D*-value and different judgment conditions, **(B)** *D*-value and different pattern arrangements, and **(C)** judgment and randomization conditions (error bars represent standard error).

**FIGURE 7:**
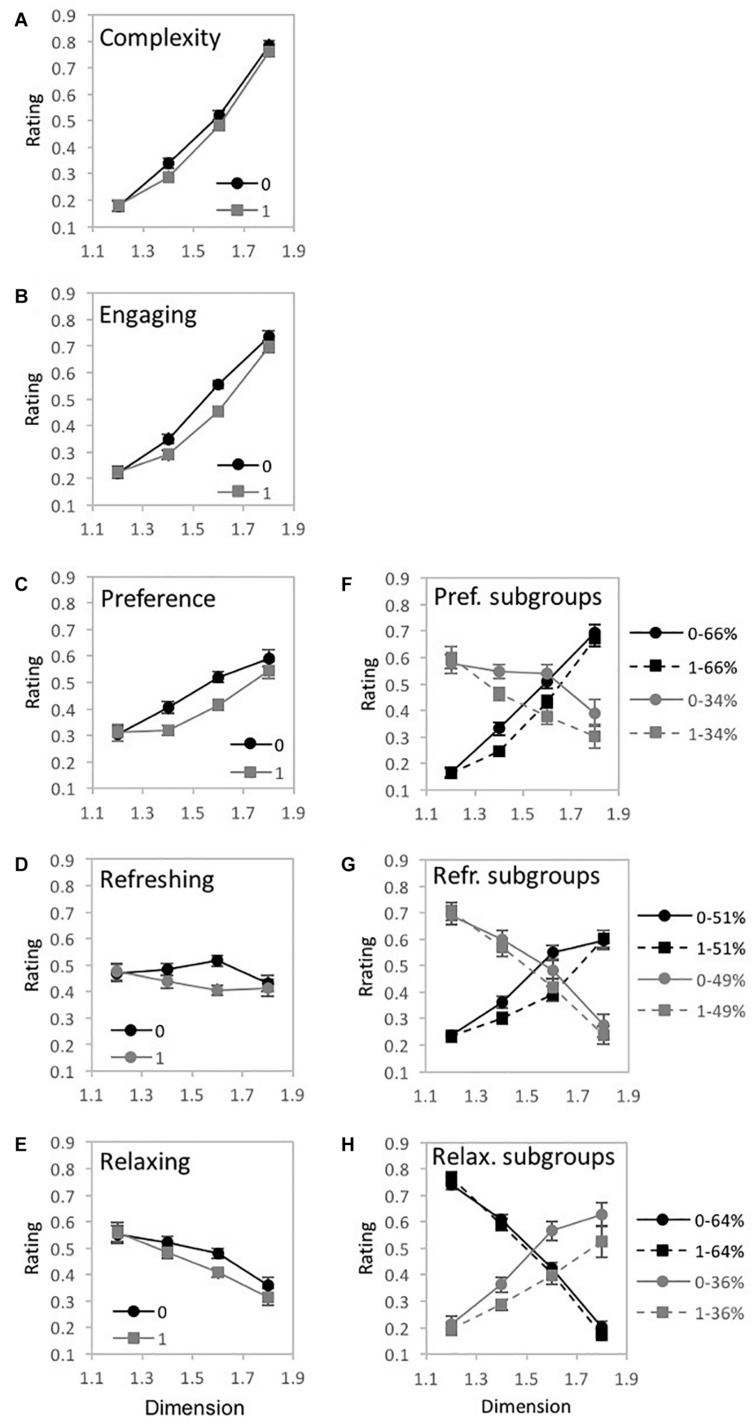
Experiment 1A results for “global forest” fractal patterns for 5 different judgment conditions (how complex, engaging, preferred, refreshing, and relaxing). **(A–E)** shows plots of mean ratings as a function of fractal dimension (*D*) and 2 pattern arrangements (not randomized “0,” randomized “1”) for the different judgment conditions (error bars represent standard error). **(F–H)** shows plots of mean ratings as a function of fractal dimension (*D*) and 2 pattern arrangements (not randomized “0,” randomized “1”) for each subpopulation identified with cluster analysis (error bars represent standard error).

A 3-way repeated measures 4 × 5 × 2 ANOVA [*D*-value (1.2, 1.4, 1.6, and 1.8) × Judgment (complexity, engaging, preference, refreshing, and relaxing) × Arrangement (randomized, non-randomized)] was performed using IBM SPSS Statistics for Macintosh (Version 25.0) on rating data for the fractal patterns (recorded as the location selected on a rating response slider), with *D*-value, Judgment, and Arrangement as within-subjects variables Mauchly’s test indicated a violation of the assumption of sphericity for *D*-value [χ^2^(5) = 160.41, *p* < 0.001^∗∗^], the interaction between *D*-value and Arrangement [χ^2^(5) = 37.92, *p* < 0.001^∗∗^], *D*-value and Judgment [χ^2^(77) = 510.44, *p* < 0.001^∗∗^], as well as the three-way interaction between *D*-value, Arrangement, and Judgment [χ^2^(77) = 134.45, *p* < 0.001^∗∗^]. Therefore, degrees of freedom were corrected using Greenhouse-Geisser estimates of sphericity (ε = 0.408, 0.679, 0.365, and 0.672, respectively). Indicated with a double asterisk for significance of *p* < 0.001 and single asterisk for significance of *p* < 0.05, a significant main effect of *D*-value [*F*(1.22, 61.2) = 23.84, *p* < 0.001^∗∗^, 95% CI [0.15, 0.23], η_*p*_^2^ = 0.32] and Arrangement emerged [*F*(1, 50) = 19.67, *p* < 0.001^∗∗^, 95% CI [0.09, 0.45], η_*p*_^2^ = 0.28]. Additional significant interactions were detected between *D*-value and Judgment [*F*(4.38, 219.07) = 55.42, *p* < 0.001^∗∗^, 95% CI [0.43, 0.59], η_*p*_^2^ = 0.53], *D*-value and Arrangement [*F*(2.04, 101.86) = 11.37, *p* < 0.001^∗∗^, 95% CI [0.06, 0.31], η_*p*_^2^ = 0.19], Arrangement and Judgment [*F*(3.47,173.45) = 2.15, *p* = 0.04^∗^, 95% CI [0.0, 0.1], η_*p*_^2^ = 0.09], as well as *D*-value, Arrangement, and Judgment [*F*(8.06, 403.14) = 1.86, *p* = 0.02^∗^, 95% CI [0.0, 0.06], η_*p*_^2^ = 0.33]. For illustrative purposes we plot the 3 significant interactions (Figure 6). For the *D*-value and Judgment interaction, some judgments had ratings that increased in value with D (complexity, engagement, and preference), while others were relatively flat (refreshing) or decreased (relaxing) (Figure 6A). For the *D*-value and Arrangement interaction, ratings were slightly higher for non-randomized fractal patterns with mid-range *D*-values (Figure 6B). Finally, for the Judgment and Arrangement interaction, the amount of difference between the non-randomized and randomized versions of the patterns varied across judgment type (Figure 6C). The 3-way interaction indicates that the Dimension by Arrangement interaction varies across Judgment-type. This can be seen more clearly in Figure 7. Below we present a series of planned analyses exploring the interaction between *D*-value and pattern Arrangement for different Judgment types in more detail using ANOVAs, paired *t*-tests (Table 1), and a 2-step cluster analysis to determine if subgroups could better explain perceptual trends.

**TABLE 1:** Experiment 1A-paired samples *t*-tests across *D*-value and judgment.

	Complex	Engaging	Preference	Refreshing	Relaxing
D = 1.2 vs. D = 1.4	t = −14.31** (d = 0.76)	t = −8.19** (d = 0.66)	t = −3.31* (d = 0.23)	t = 0.71 (d = 0.37)	t = 3.57** (d = 0.24)
D = 1.2 vs. D = 1.6	t = −23.26** (d = 2.17)	t = −15.79* (d = 1.7)	t = −6.12** (d = 0.75)	t = 0.42 (d = 0.05)	t = 3.88** (d = 0.51)
D = 1.2 vs. D = 1.8	t = −28.47** (d = 3.56)	t = −20.56** (d = 2.77)	t = −6.86** (d = 1.02)	t = 1.24 (d = 0.19)	T = 5.16** (d = 0.79)
D = 1.4 vs. D = 1.6	t = −16.51** (d = 1.23)	t = −14.68** (d = 1.2)	t = −6.04** (d = 0.63)	t = 0.01 (d = 0.0)	t = 3.17* (d = 0.34)
D = 1.4 vs. D = 1.8	t = −24.42** (d = 2.97)	t = −20.5** (d = 2.4)	t = −6.76** (d = 1.2)	t = 1.25 (d = 0.17)	t = 5.07** (d = 0.69)
D = 1.6 vs. D = 1.8	t = −18.38** (d = 1.8)	t = −13.98** (d = 1.25)	t = −5.03** (d = 0.59)	t = 1.91 (d = 0.17)	t = 5.48** (d = 0.45)

*Indicates significance of p < 0.05. **Indicates significance of p < 0.001.

##### Complexity

A 2-way 4 × 2 repeated-measures ANOVA [*D*-value (1.2, 1.4, 1.6, and 1.8) × Arrangement (randomized, non-randomized)] was completed to examine the impact of *D-*value and Arrangement on pattern complexity judgments (Figure 7A). Assumptions of the violation of sphericity were indicated by Mauchly’s test for *D*-value [χ^2^(5) = 123.06, *p* < 0.001^∗∗^] and the interaction between *D*-value and Arrangement [χ^2^(5) = 15.66, *p* = 0.01^∗^], thus degrees of freedom were corrected using Greenhouse-Geisser estimates of sphericity (ε = 0.512 and 0.87, respectively). A significant main effect of *D*-value [*F*(1.54, 118.21) = 343.44, *p* < 0.001^∗∗^, 95% *CI* [0.76, 0.85], η_*p*_^2^ = 0.82], Arrangement [*F*(1,77) = 10.63, *p* = 0.002^∗^, 95% *CI* [0.02, 0.26], η_*p*_^2^ = 0.12], and interaction between *D*-value and pattern arrangement [*F*(2.6,200.94) = 2.99, *p* = 0.04^∗^, 95% *CI* [0.0, 0.07], η_*p*_^2^ = 0.04] were identified. Average complexity ratings (collapsed over pattern arrangement type) ranged from a low of 0.18 (*SD* = 0.18) for *D* = 1.2 to a high of 0.77 (*SD* = 0.15) for *D* = 1.8, indicating that participants perceived greater complexity for patterns with higher *D*-values. Paired samples *t*-tests revealed significant differences in perceived complexity between all pairs of *D*-values (Table 1). When comparing non-random and random pattern arrangements, significant differences exist for the mid-range *D*-values: *D* = 1.4 [*t*(77) = 3.79, *p* < 0.001^∗∗^, 95% *CI* [0.02, 0.08], *d* = 0.32] and *D* = 1.6 [*t*(77) = 2.31, *p* = 02^∗^, 95% *CI* [0.01, 0.07], *d* = 0.28]. The interaction between *D*-value and Arrangement indicates that the ratings differed across arrangement type depending on *D*-value, with slightly higher ratings for non-randomized compared to randomized fractal patterns with mid-range *D*-values.

To determine whether the observed trends could be due to a combination of responses from subgroups of participants, we performed a two-step cluster analysis similar to that used by Bies et al. (2016) and described in more detail in Norus̆is (2012). We performed a hierarchical cluster analysis using Ward’s method to separate individuals into groups using their complexity ratings for each level of *D*. Since the resultant agglomeration matrix did not indicate a multiple cluster solution, we did not follow up with a *k*-means clustering analysis.

##### Engaging

A 2-way 4 × 2 repeated-measures ANOVA [*D*-value (1.2, 1.4, 1.6, and 1.8) × Arrangement (randomized, non-randomized)] was completed to examine the impact of *D-*value and Arrangement on pattern engagement (Figure 7B). A violation of the assumption of sphericity was indicated by Mauchly’s test for *D*-value [χ^2^(5) = 114.57, *p* < 0.001^∗∗^], thus degrees of freedom were corrected using Greenhouse-Geisser estimates of sphericity (ε = 0.521). A significant main effect of *D*-value [*F*(1.56, 114.01) = 194.5, *p* < 0.001^∗∗^, 95% *CI* [0.63, 0.78], η_*p*_^2^ = 0.73], Arrangement [*F*(1,73) = 19.69, *p* < 0.001^∗∗^, 95% *CI* [0.07, 0.36], η_*p*_^2^ = 0.21], and significant interaction between *D*-value and Arrangement [*F*(2.71,198.09) = 9.58, *p* = 0.04^∗^, 95% *CI* [04, 0.19], η_*p*_^2^ = 0.12] were identified. Collapsed over pattern arrangement, the mean engagement ratings ranged from a low of 0.22 (*SD* = 0.17) for *D* = 1.2 to a high of 0.72 (*SD* = 0.19) for *D* = 1.8, suggesting that participants were more engaged when viewing the higher *D*-value patterns. Paired samples *t*-tests revealed significant differences in perceived engagement for all pairs of *D*-values (Table 1). Comparing the non-random and random arrangements for different *D*-values, significant differences exist for the mid-range *D*-values: *D* = 1.4 [*t*(73) = −4.12, *p* < 0.001^∗∗^, 95% *CI* [−0.09, −0.04], *d* = 0.44] and *D* = 1.6 [*t*(73) = −4.95, *p* < 0.001^∗∗^, 95% *CI* [−0.14, −0.06], *d* = 0.73]. Again, the interaction between *D*-value and Arrangement indicates that the ratings differed across arrangement type depending on *D*-value, with slightly higher ratings for non-randomized compared to randomized fractal patterns with mid-range *D*-values. A cluster analyses did not indicate a multiple cluster solution.

##### Preference

A 2-way 4 × 2 repeated-measures ANOVA [*D*-value (1.2, 1.4, 1.6, and 1.8) × Arrangement (randomized, non-randomized)] was completed to examine the impact of *D-*value and Arrangement on pattern preference (Figure 7C). A violation of the assumption of sphericity was indicated by Mauchly’s test for *D*-value [χ^2^(5) = 159.69, *p* < 0.001^∗∗^] and the interaction between *D-*value and Arrangement [χ^2^(5) = 23.54, *p* < 0.001^∗∗^], thus degrees of freedom were corrected using Greenhouse-Geisser estimates of sphericity (ε = 0.445 and 0.795, respectively). A significant main effect of *D*-value [*F*(1.33,89.38) = 23.27, *p* < 0.001^∗∗^, 95% *CI* [0.11, 0.39], η_*p*_^2^ = 0.26], Arrangement [*F*(1,67) = 18, *p* < 0.001^∗∗^, 95% *CI* [0.06, 0.37], η_*p*_^2^ = 0.21], and interaction between *D*-value and Arrangement were identified [*F*(2.39,159.84) = 6.25, *p* = 0.001^∗^, 95% *CI* [0.01, 0.17], η_*p*_^2^ = 0.09]. Collapsed over pattern arrangement, average ratings of preference ranged from a low of 0.31 (*SD* = 0.25) for *D* = 1.2 to a high of 0.57 (*SD* = 0.26) for *D* = 1.8, indicating that participants’ preference for global forest fractals increases with pattern complexity. Paired samples *t*-tests revealed significant differences in preference for all pairs of *D*-values (Table 1). Comparing non-random and random arrangements, significant differences exist for the mid-range *D*-values: *D* = 1.4 [*t*(67) = 5.40, *p* < 0.001^∗∗^, 95% *CI* [0.05, 0.11], *d* = 0.47] and *D* = 1.6 [*t*(67) = 4.45, *p* < 0.001^∗∗^, 95% *CI* [0.06, 0.15], *d* = 0.65].

A 2-step cluster analysis identified and separated individuals into 2 subgroups (Figure 7F). We investigated whether there was an interaction between cluster-membership, *D*-value, and arrangement by performing a mixed ANOVA with 4 levels of *D*, 2 levels of arrangement, and 2 groups. Mauchly’s test indicated a violation of the assumptions of sphericity for *D*-value [χ^2^(5) = 58.05, *p* < 0.001^∗∗^] and the interaction between *D*-value and arrangement [χ^2^(5) = 21.95 *p* = 0.001^∗^]. Therefore, degrees of freedom were corrected using Greenhouse-Geisser estimates of sphericity (ε = 0.676 and 0.805, respectively). A significant main effect of *D*-value [*F*(2.03,133.78) = 16.01, *p* < 0.001^∗∗^, 95% *CI* [0.08, 0.3], η_*p*_^2^ = 0.2] and Arrangement emerged in the analysis [*F*(1,66) = 18.88, *p* < 0.001^∗∗^, 95% *CI* [0.07, 0.38], η_*p*_^2^ = 0.22], as well as a significant interaction between *D*-value and Cluster groups [*F*(2.03,133.78) = 105.22, *p* < 0.001^∗∗^, 95% *CI* [0.51, 0.68], η_*p*_^2^ = 0.62] as well as *D*-value and Arrangement [*F*(2.41,159.32) = 7.12, *p* < 0.001^∗∗^, 95% *CI* [0.02, 0.18], η_*p*_^2^ = 1.0]. The first cluster accounts for 66% of the sample and is most reflective of the overall perceptual trend with preference ratings increasing with higher *D*-value. The second cluster includes the remaining 34% of the sample and demonstrates an opposing trend with preference peaking with lower *D*-value and decreasing with added complexity. Although, on average, preference is highest for *D* = 1.8 (Figure 7C), the subgroup analysis shows that the *D*-value with the greatest agreement in preference amongst individuals in the different subgroups is *D* = 1.6 (Figure 7F).

##### Refreshing

A 2-way 4 × 2 repeated-measures ANOVA [*D*-value (1.2, 1.4, 1.6, and 1.8) × Arrangement (randomized, non-randomized)] was completed to examine the impact of *D-*value and Arrangement on perceived pattern refreshment (Figure 7D). A violation of the assumption of sphericity was indicated by Mauchly’s test for *D*-value [χ^2^(5) = 213.96, *p* < 0.001^∗∗^], thus degrees of freedom were corrected using Greenhouse-Geisser estimates of sphericity (ε = 0.399). Both the main effect of pattern Arrangement [*F*(1,71) = 11.66, *p* = 0.001^∗^, 95% *CI* [0.02, 0.29], η_*p*_^2^ = 0.14] and interaction between *D*-value and Arrangement were significant [*F*(2.72,193.37) = 7.95, *p* < 0.001^∗∗^, 95% *CI* [0.03, 0.18], η_*p*_^2^ = 0.1], but not *D*-value itself [*F*(1.2,84.89) = 0.77, *p* = 0.41, 95% *CI* [0.0, 0.09] η_*p*_^2^ = 0.01]. Between non-random and random arrangements, significant differences exist for the mid-range *D*-values: *D* = 1.4 [*t*(71) = 2.79, *p* = 0.01^∗^, 95% *CI* [0.01, 0.08], *d* = 0.2] and *D* = 1.6 [*t*(71) = 4.54, *p* < 0.001^∗∗^, 95% *CI* [0.06, 0.16], *d* = 0.67].

A 2-step cluster analysis identified and separated individuals into two subgroups with respect to ratings of pattern refreshment (Figure 7G). We investigated whether there was an interaction between cluster-membership, *D*-value, and arrangement by performing a mixed ANOVA with 4 levels of *D*, 2 levels of arrangement, and 2 groups. Mauchly’s test indicated a violation of the assumptions of sphericity for *D*-value [χ^2^(5) = 73.86, *p* < 0.001^∗∗^] and interaction between *D*-value and Arrangement [χ^2^(5) = 11.49, *p* = 0.04^∗^]. Therefore, degrees of freedom were corrected using Greenhouse-Geisser estimates of sphericity (ε = 0.580 and 0.893, respectively). Whereas the main effect of *D*-value was not significant [*F*(1.74,121.72) = 1.49, *p* = 0.23, 95% *CI* [0.0, 0.09], η_*p*_^2^ = 0.02], a significant main effect of Arrangement emerged in the analysis [*F*(1,70) = 11.85, *p* = 0.001^∗^, 95% *CI* [0.03, 0.29], η_*p*_^2^ = 0.15], as well as a significant interaction between *D*-value and Cluster membership [*F*(1.74,121.72) = 143.09, *p* < 0.001^∗∗^, 95% *CI* [0.57, 0.73], η_*p*_^2^ = 0.67] and between *D*-value and Arrangement [*F*(2.68,187.57) = 8.32, *p* < 0.001^∗∗^, 95% *CI* [0.03, 0.18], η_*p*_^2^ = 0.11]. The first cluster encompassed 51% of participants and produces a trend that increases with *D*-value. The second cluster contains the remaining 49% of participants and, in a steeper fashion, decreases with additional *D*-value. Although these represent opposing trends in judgments of refreshment, the *D*-value with the greatest agreement in refreshment ratings amongst individuals across subgroups is *D* = 1.6 (Figure 7G).

##### Relaxing

A 2-way 4 × 2 repeated-measures ANOVA [*D*-value (1.2, 1.4, 1.6, and 1.8) × Arrangement (randomized, non-randomized)] was completed to examine the impact of *D-*value and Arrangement on perceptions of pattern relaxation (Figure 7E). A violation of the assumption of sphericity was indicated by Mauchly’s test for *D*-value [χ^2^(5) = 239.32 *p* < 0.001^∗∗^], thus degrees of freedom were corrected using Greenhouse-Geisser estimates of sphericity (ε = 0.388). A significant main effect of *D*-value [*F*(1.16,79.11) = 11.9, *p* < 0.001^∗∗^, 95% CI [0.03, 0.29], η_*p*_^2^ = 0.15] and Arrangement [*F*(1,68) = 8.19, *p* = 0.01^∗^, 95% CI [0.01, 0.25], η_*p*_^2^ = 0.11], and interaction between *D*-value and pattern arrangement were identified [*F*(2.74,186.02) = 4.7, *p* = 0.01^∗^ 95% CI [0.01, 0.13], η_*p*_^2^ = 0.01]. Collapsed over pattern arrangement, average ratings of pattern relaxation ranged from a low of 0.34 (SD = 0.27) for *D* = 1.8 to a high of 0.56 (SD = 0.29) for D = 1.2, suggesting that participants perceived patterns as less relaxing with increasing *D*-value. Paired samples *t*-tests revealed significant differences in perceived relaxation between *D*-values (see Table 1). Comparing non-random and random arrangements, significant differences exist for the mid- to high-D patterns: D = 1.4 [*t*(68) = 2.22, *p* < 0.001^∗∗^, 95% CI [0.0, 0.07], *d* = 0.21], *D* = 1.6 [*t*(68) = 3.15, *p* = 002^∗^, 95% CI [0.03, 0.12], *d* = 0.5], and *D* = 1.8 [*t*(68) = 2.18, *p* = 0.03^∗^, 95% CI [0.0, 0.1], *d* = 0.22].

A 2-step cluster analysis identified and separated individuals into two subgroups with respect to ratings of pattern relaxation. Mauchly’s test indicated a violation of the assumptions of sphericity for *D*-value [χ^2^(5) = 93.78, *p* < 0.001^∗∗^]. Therefore, degrees of freedom were corrected using Greenhouse-Geisser estimates of sphericity (ε = 0.554). A significant main effect of *D*-value [*F*(1.66,111.27) = 7.28, *p* = 0.002^∗^, 95% *CI* [0.01, 0.2], η_*p*_^2^ = 0.1] and Arrangement [*F*(1,67) = 13.86, *p* < 0.001^∗∗^, 95% *CI* [0.04, 0.33], η_*p*_^2^ = 0.17] were identified, as well as significant interactions between *D*-value and Clusters [*F*(1.66,111.27) = 168.83, *p* < 0.001^∗∗^, 95% *CI* [0.62, 0.77], η_*p*_^2^ = 0.72], Arrangement and Cluster membership [*F*(1,67) = 8.81, *p* = 0.004^∗^, 95% *CI* [0.01, 0.26], η_*p*_^2^ = 0.12], and *D*-value and Arrangement [*F*(2.72,181.88) = 5.79, *p* = 0.001^∗^, 95% *CI* [0.01, 0.15], η_*p*_^2^ = 0.08]. The first cluster encompassed 64% of participants and produces a trend in which ratings of pattern relaxation steeply decrease with higher *D*-values. Conversely, the second cluster contains the remaining 36% of participants and increases with additional *D*-value. Similar to subgroup behavior for preference and refreshment ratings which also showed opposing trends in judgments, the *D*-value with the greatest agreement in relaxation ratings amongst individuals across subgroups is *D* = 1.6 (Figure 7H).

#### Discussion

Experiment 1A explored broad psychological effects of fractal patterns used in installations of multiple mediums including carpets, wall patterns, and screensavers. Overall, we find that perceptions of fractal pattern complexity, engagement, and preference, increase with greater *D*-value, perception of pattern refreshment is unchanging across *D*-value, and perception of relaxation decreases with *D*-value. For some judgments, the observed overall trends can be explained by the subgroup patterns of responses. We found 2 subgroups for preference, refreshment, and relaxation judgments with opposing trends. The overall trend for preference was positive, with increasing rating values with increasing *D*-value, because the largest subgroup trend was positive; the trend for refreshing was flat because the 2 subgroups were equivalent in size; and, finally, the overall trend for relaxation was negative (decreasing with *D*-value) because the largest subgroup trend was negative. Interestingly, the *D*-value with the greatest agreement amongst individuals for the preference, refreshing, and relaxing judgments was *D* = 1.6.

### Experiment 1B – Perception of Fractal “Global Forest” Patterns With Bipolar Ratings

#### Materials and Methods

##### Stimuli

The current experiment used the same stimuli as described in Experiment 1A.

##### Participants

81 participants (69 females), comprised of undergraduate Psychology students from the UNSW Sydney volunteered to participate in the current study through the SONA participant pool system in exchange for course credit. The mean age of participants was 20.42 years (ranging between 18 and 47 years). All study protocols, including obtaining Informed Consent were approved by the UNSW Human Research Advisory Panel (Reference ID: HREAP-C 2349).

##### Visual displays

The study was generated with Inquisit (by Milliseconds) software and run via the Inquisit Web Platform. The participants completed the study on their personal computers with program stimuli scaled to the individual computer’s respective full-screen dimensions.

##### Design and procedure

Like in the Experiment 1A, participants viewed the “global forest” fractal patterns presented in separate randomized blocks, with each block consisting of a singular judgment type (complexity, engaging, preference, refreshing, or relaxing). Each block’s stimulus set consisted of 4 unique patterns ranging across eight levels of complexity or *D*-value (1.1, 1.2, 1.3, 1.4, 1.5, 1.6, 1.7, and 1.8) and varying in arrangement (non-randomized or randomized) giving rise to 64 trials per block and 320 total stimulus-related trials across the experiment. Instead of a slider-type response, we used five, Lickert-type, bipolar scales with values ranging from 1 to 7. The scales used were simple-complex; dislike (1) -like (7); indifferent (1) – engaged (7); relaxed (1) – tense (7); tired (1) – refreshed (7). [BS1] Participants indicated their response by pressing a number corresponding to their evaluation of a given pattern. Before each block, participants were introduced to a scale that will be used in that block, with the scale remaining visible on all trials. Upon completion of the experiment, participants completed a demographic questionnaire and were debriefed according to the protocol approved by the UNSW Human Research Ethics Advisory Panel C.

#### Results

Data from 75 adult participants were analyzed with 6 participants excluded due to a failure to complete the study (4 participants), or technical error with data recording (2 participants).

##### Fractal judgment task

**FIGURE 8:**
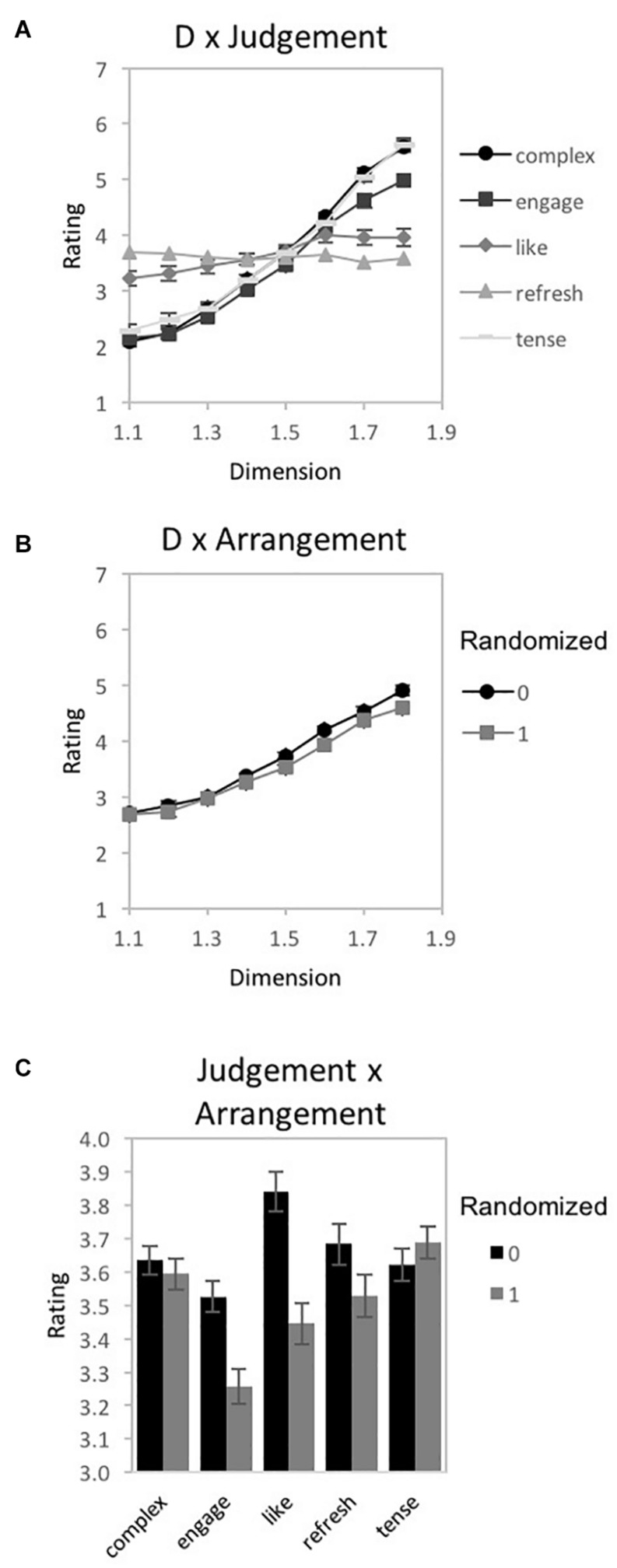
Experiment 1B. Results for “global forest” fractal patterns using a bipolar rating scale. Results show significant 2-way interactions among the experiment’s 3 factors: fractal dimension (*D*), stimulus pattern arrangement (“0” for non-randomized and “1” for randomized), and judgment type (complex, engaging, liking, refreshing, and tense). Participant rating (on a scale from 1 to 10) is plotted as a function of **(A)** *D*-value and different judgment conditions, **(B)** *D*-value and different pattern arrangements, and **(C)** judgment and randomization conditions (error bars represent standard error).

**FIGURE 9:**
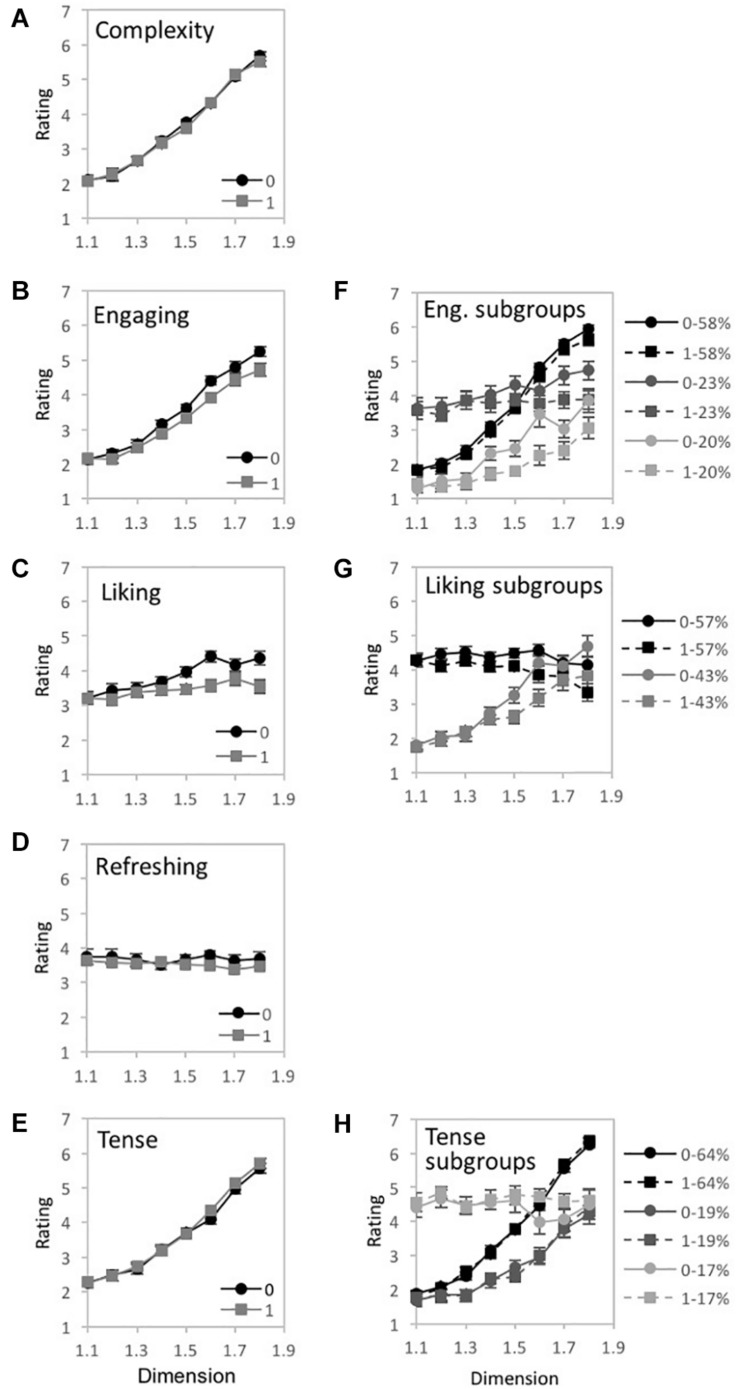
Experiment 1B results for “global forest” fractal patterns for 5 different judgment conditions (complex, engaging, liking, refreshing, and tense). **(A–E)** shows plots of mean ratings as a function of fractal dimension (*D*) and 2 pattern arrangements (not randomized “0,” randomized “1”) for the different judgment conditions (error bars represent standard error). **(F–H)** shows plots of mean ratings as a function of fractal dimension (*D*) and 2 pattern arrangements (not randomized “0,” randomized “1”) for each subpopulation identified with cluster analysis (error bars represent standard error).

A 3-way 8 × 5 × 2 repeated-measures ANOVA [*D*-value (1.1, 1.2, 1.3, 1.4, 1.5, 1.6, 1.7, and 1.8) × Judgment (complexity, engaging, liking, refreshing, and tense) × Arrangement (randomized vs. non-randomized)] was performed using IBM SPSS Statistics for Macintosh (Version 25.0) on rating data for the fractal patterns (recorded as location selected on a rating response slider). Mauchly’s test indicated a violation of the assumption of sphericity for *D*-value [χ^2^(27) = 638.83, *p* < 0.001^∗∗^], Judgment [χ^2^(9) = 39.25, *p* < 0.001^∗∗^], the interaction between *D*-value and Arrangement [χ^2^(27) = 63.65, *p* < 0.001^∗∗^], *D*-value and Judgment [χ^2^(405) = 2571.82, *p* < 0.001^∗∗^], Judgment and Arrangement [χ^2^(9) = 64.32, *p* < 0.001^∗∗^], as well as the three-way interaction between *D*-value, Arrangement, and Judgment [χ^2^(405) = 647.13, *p* < 0.001^∗∗^]. Therefore, degrees of freedom were corrected using Greenhouse-Geisser estimates of sphericity (ε = 0.205, 0.789, 0.749, 0.121, 0.692, and 0.542, respectively). Indicated with a double asterisk for significance of *p* < 0.001 and single asterisk for statistical significance of *p* < 0.05, a significant main effect of *D*-value [*F*(1.44,106.27) = 153.44, *p* < 0.001^∗∗^, 95% *CI* [0.57, 0.74], η_*p*_^2^ = 0.68] and Arrangement emerged [*F*(1,74) = 31.44, *p* < 0.001^∗∗^, 95% *CI* [0.3, 0.44], η_*p*_^2^ = 0.3]. Additional significant interactions were found between *D*-value and Judgment [*F*(3.38,249.96) = 32.35, *p* < 0.001^∗∗^, 95% *CI* [0.21, 0.38], η_*p*_^2^ = 0.3], *D*-value and Arrangement [*F*(5.25, 388.12) = 4.95, *p* < 0.001^∗∗^, 95% *CI* [0.02, 0.10], η_*p*_^2^ = 0.06], Arrangement and Judgment [*F*(2.78,204.98) = 9.59, *p* < 0.001^∗∗^, 95% *CI* [0.04, 0.09], η_*p*_^2^ = 0.12], as well as *D*-value, Arrangement, and Judgment [*F*(15.18,1123.58) = 2.75, *p* < 0.001^∗∗^, 95% *CI* [0.01, 0.05], η_*p*_^2^ = 0.04]. For illustrative purposes we plot the 3 significant interactions (Figure 8). For the *D*-value and Judgment interaction, most judgments had ratings that increased in value with *D* (complexity, engagement, like, and tense), while one was relatively flat (refreshing) (Figure 8A). For the *D*-value and Arrangement interaction, ratings were increasingly higher for non-randomized compared to randomized fractal patterns as *D*-values increased (Figure 8B). Finally, for the Judgment and Arrangement interaction, the amount of difference between the non-randomized and randomized versions of the patterns varied across judgment type (Figure 8C). The 3-way interaction indicates that the Dimension by Arrangement interaction varies across Judgment-type. This can be seen more clearly in Figure 9. Similar to the previous studies, a series of planned comparisons explored the locus of the significant interaction between *D*-value and Judgment through ANOVAs, paired *t*-tests (Table 2), and a 2-step cluster analyses to determine if subgroups of participant responses could better explain perceptual trend data.

**TABLE 2:** Experiment 1B- Paired Samples *t*-tests across *D*-value and Judgment.

	Simple-Complex	Indifferent-Engaging	Dislike-Like	Tired-Refreshing	Relaxing-Tense
D = 1.1 vs. D = 1.2	t = −3.43* (d = 1.3)	t = −1.93** (d = 0.93)	t = −1.35 (d = 0.17)	t = 0.39 (d = 0.01)	t = −3.95** (d = 0.15)
D = 1.1 vs. D = 1.3	t = −10.05** (d = 0.51)	t = −16.01** (d = 0.32)	t = −3.03* (d = 1.42)	t = 0.99 (d = 0.04)	t = −6.63** (d = 0.34)
D = 1.1 vs. D = 1.4	t = −14.18** (d = 1.02)	t = −10.61** (d = 0.8)	t = −3.51* (d = 0.25)	t = 1.10 (d = 0.08)	t = −11.23** (d = 0.78)
D = 1.1 vs. D = 1.5	t = −18.17** (d = 1.51)	t = −13.01** (d = 1.1)	t = −4.32** (d = 0.36)	t = 0.58 (d = 0.06)	t = −12.84** (d = 1.18)
D = 1.1 vs. D = 1.6	t = −19.81** (d = 2.09)	t = −14.87** (d = 1.7)	t = −4.82** (d = 0.52)	t = 0.19 (d = 0.08)	t = −14.55** (d = 1.61)
D = 1.1 vs. D = 1.7	t = −21.02** (d = 2.61)	t = −16.62** (d = 1.9)	t = −4.05** (d = 0.47)	t = 0.72 (d = 0.10)	t = −17.25** (d = 2.25)
D = 1.1 vs. D = 1.8	t = −24.54** (d = 3.03)	t = −17.54** (d = 2.14)	t = −3.48** (d = 0.44)	t = 0.38 (d = 0.06)	t = −18.89** (d = 2.61)
D = 1.2 vs. D = 1.3	t = −6.51** (d = 0.35)	t = −4.72** (d = 1.15)	t = −1.71 (d = 0.08)	t = 0.78 (d = 0.03)	t = −3.52* (d = 0.17)
D = 1.2 vs. D = 1.4	t = −11.02** (d = 0.83)	t = −11.13** (d = 1.71)	t = −2.75* (d = 0.18)	t = 1.04 (d = 0.06)	t = −9.41** (d = 0.61)
D = 1.2 vs. D = 1.5	t = −15.53** (d = 1.28)	t = −13.45** (d = 2.06)	t = −3.69** (d = 0.3)	t = 0.46 (d = 0.04)	t = −12.04** (d = 1.0)
D = 1.2 vs. D = 1.6	t = −16.89** (d = 1.83)	t = −15.58** (d = 2.55)	t = −4.51** (d = 0.47)	t = 0.08 (d = 0.01)	t = −13.53** (d = 1.43)
D = 1.2 vs. D = 1.7	t = −18.69** (d = 2.36)	t = −17.18** (d = 1.68)	t = −3.65** (d = 0.42)	t = 0.67 (d = 0.09)	t = −16.21** (d = 2.07)
D = 1.2 vs. D = 1.8	t = −21.79** (d = 2.75)	t = −18.29** (d = 2.9)	t = −3.19* (d = 0.39)	t = 0.31 (d = 0.04)	t = −18.15** (d = 2.43)
D = 1.3 vs. D = 1.4	t = −7.79** (d = 0.52)	t = −7.0** (d = 0.45)	t = −1.4 (d = 0.10)	t = 0.57 (d = 0.03)	t = −7.62** (d = 0.46)
D = 1.3 vs. D = 1.5	t = −14.22* (d = 1.01)	t = −11.16** (d = 0.82)	t = −2.83* (d = 0.21)	t = 0.06 (d = 0.01)	t = −11.44** (d = 0.89)
D = 1.3 vs. D = 1.6	t = −16.31** (d = 1.61)	t = −13.35** (d = 1.37)	t = −3.75** (d = 0.39)	t = −0.37 (d = 0.03)	t = −14.24** (d = 1.35)
D = 1.3 vs. D = 1.7	t = −19.33** (d = 2.19)	t = −15.86** (d = 1.61)	t = −3.06* (d = 0.34)	t = 0.48 (d = 0.33)	t = −17.24** (d = 2.03)
D = 1.3 vs. D = 1.8	t = −22.58** (d = 2.51)	t = −16.61** (d = 1.84)	t = −2.64* (d = 0.32)	t = 0.11 (d = 0.02)	t = −19.23** (d = 2.42)
D = 1.4 vs. D = 1.5	t = −7.38** (d = 0.49)	t = −6.59** (d = 0.41)	t = −2.01 (d = 0.13)	t = −0.50 (d = 0.02)	t = −6.49** (d = 0.45)
D = 1.4 vs. D = 1.6	t = −12.26** (d = 1.4)	t = −11.68** (d = 1.01)	t = −3.79** (d = 0.33)	t = −0.78 (d = 0.08)	t = −10.29** (d = 0.92)
D = 1.4 vs. D = 1.7	t = −16.81** (d = 1.77)	t = −13.51** (d = 1.28)	t = −2.78* (d = 0.28)	t = 0.30 (d = 0.04)	t = −14.57** (d = 1.63)
D = 1.4 vs. D = 1.8	t = −20.82** (d = 2.21)	t = −15.08** (d = 1.54)	t = −2.32* (d = 0.26)	t = −0.11 (d = 0.01)	t = −17.68** (d = 2.04)
D = 1.5 vs. D = 1.6	t = −8.49** (d = 0.67)	t = −8.47** (d = 0.61)	t = −2.64* (d = 0.21)	t = −0.59 (d = 0.05)	t = −6.11** (d = 0.47)
D = 1.5 vs. D = 1.7	t = −14.74** (d = 0.67)	t = −12.28** (d = 0.91)	t = −1.95 (d = 0.17)	t = 0.74 (d = 0.07)	t = −13.35** (d = 1.18)
D = 1.5 vs. D = 1.8	t = −19.12** (d = 1.83)	t = −13.59** (d = 1.67)	t = −1.58 (d = 0.18)	t = 0.13 (d = 0.01)	t = −17.27** (d = 1.61)
D = 1.6 vs. D = 1.7	t = −10.34** (d = 0.75)	t = −5.87** (d = 0.35)	t = 0.38 (d = 0.02)	t = 1.69 (d = 0.11)	t = −10.3** (d = 0.73)
D = 1.6 vs. D = 1.8	t = −17.44** (d = 1.2)	t = −9.82** (d = 0.6)	t = 0.33 (d = 0.02)	t = 0.57 (d = 0.05)	t = −15.23** (d = 1.18)
D = 1.7 vs. D = 1.8	t = −7.99** (d = 0.41)	t = −5.86** (d = 0.25)	t = 0.04 (d = 0.0)	t = −0.90 (d = 0.04)	t = −9.9** (d = 0.47)

*Indicates significance of p < 0.05. **Indicates significance of p < 0.001.

##### Simple-complex

A 2-way 8 × 2 repeated-measures ANOVA [*D*-value (1.1, 1.2, 1.3, 1.4, 1.5, 1.6, 1.7, and 1.8) × Arrangement (randomized vs. non-randomized)] was completed to examine the impact of *D-*value and Arrangement on pattern complexity judgments (Figure 9A). Assumptions of the violation of sphericity were indicated by Mauchly’s test for *D*-value [χ^2^(27) = 521.02, *p* < 0.001^∗∗^] and interaction between *D*-value and Arrangement [χ^2^(27) = 76.63, *p* < 0.001^∗∗^], thus degrees of freedom were corrected using Greenhouse-Geisser estimates of sphericity (ε = 0.232 and 0.764, respectively). A significant main effect of *D*-value was identified [*F*(1.62,119.95) = 204.35, *p* < 0.001^∗∗^, 95% CI [0.65, 0.79], η_*p*_^2^ = 0.73], however, no significant effect of Arrangement [*F*(1,74) = 0.89, *p* = 0.35, 95% CI [0, 0.1], η_*p*_^2^ = 0.01], nor interaction between *D*-value and pattern arrangement [*F*(5.35,395.78) = 0.79, *p* = 0.56, 95% CI [0, 0.02], η_*p*_^2^ = 0.01] were identified. Average complexity ratings (collapsed over pattern arrangement type) ranged from a low of 2.09 (SD = 1.16) for *D* = 1.1 to a high of 5.60 (SD = 1.16) for *D* = 1.8, indicating that participants perceive greater complexity for patterns with higher *D*-values. Paired samples *t*-tests revealed significant differences in perceived refreshment between all pairs of *D*-values (Table 2). A cluster analysis did not indicate a multiple cluster solution.

##### Indifferent-engaging

A 2-way 8 × 2 repeated-measures ANOVA [*D*-value (1.1, 1.2, 1.3, 1.4, 1.5, 1.6, 1.7, and 1.8) × Arrangement (randomized vs. non-randomized)] was completed to examine the impact of *D-*value and Arrangement on pattern engagement (Figure 9B). A violation of the assumption of sphericity was indicated by Mauchly’s test for *D*-value [χ^2^(27) = 443.38, *p* < 0.001^∗∗^] and interaction of *D*-value and Arrangement [χ^2^(27) = 88.06, *p* < 0.001^∗∗^], thus degrees of freedom were corrected using Greenhouse-Geisser estimates of sphericity (ε = 0.260 and 0.661, respectively). A significant main effect of *D*-value [*F*(1.82,134.71) = 131.39, *p* < 0.001^∗∗^, 95% *CI* [0.54, 0.71], η_*p*_^2^ = 0.64], Arrangement [*F*(1,74) = 16.98, *p* < 0.001^∗∗^, 95% *CI* [0.05, 0.33], η_*p*_^2^ = 0.19], and interaction between *D*-value and pattern arrangement were identified [*F*(4.63,342.64) = 3.8, *p* = 0.003^∗^, 95% *CI* [0.01, 0.09], η_*p*_^2^ = 0.05]. Collapsed over pattern arrangement, the mean engagement ratings ranged from a low of 2.15 (*SD* = 1.15) for *D* = 1.1 to a high of 4.98 (*SD* = 1.48) for *D* = 1.8, suggesting that participants were more engaged when viewing the higher *D*-value patterns. Paired samples *t*-tests revealed significant differences in perceived engagement for all pairs of *D*-values (Table 2). Comparing the non-random and random arrangements for different *D*-values, significant differences exist for the mid- to high-range *D*-values: *D* = 1.4 [*t*(74) = 3.1, *p* = 0.003^∗^, 95% *CI* [0.1, 0.45], *d* = 0.26], *D* = 1.5 [*t*(74) = 2.68, *p* = 01^∗^, 95% *CI* [0.07, 0.48], *d* = 0.26], *D* = 1.6 [*t*(74) = 3.16, *p* = 0.002^∗^, 95% *CI* [0.18, 0.78], *d* = 0.4], *D* = 1.7 [*t*(74) = 3.05, *p* = 0.003^∗^, 95% *CI* [0.13, 0.62], *d* = 0.27], and *D* = 1.8 [*t*(74) = 3.77, *p* < 0.001^∗∗^, 95% *CI* [0.25, 0.8], *d* = 0.36]. The interaction between *D*-value and Arrangement indicates that the ratings differed across arrangement type depending on *D*-value, with increasingly higher ratings for non-randomized compared to randomized fractal patterns as *D*-values increased.

A 2-step cluster analysis identified and separated individuals into 3 subgroups (Figure 9F). Mauchly’s test indicated a violation of the assumptions of sphericity for *D*-value [χ^2^(27) = 214.33, *p* < 0.001^∗∗^] and the interaction between *D*-value and Arrangement [χ^2^(27) = 82.31, *p* < 0.001^∗∗^]. Therefore, degrees of freedom were corrected using Greenhouse-Geisser estimates of sphericity (ε = 0.446 and 0.673, respectively). A significant main effect of *D*-value [*F*(3.12,224.58) = 116.01 *p* < 0.001^∗∗^, 95% *CI* [0.54, 0.67], η_*p*_^2^ = 0.62], and Arrangement emerged in the analysis [*F*(1,72) = 22.75, *p* < 0.001^∗∗^, 95% *CI* [0.09, 0.39], η_*p*_^2^ = 0.24], as well as significant interactions between *D*-value and Clusters [*F*(6.24,224.58) = 31.25, *p* < 0.001^∗∗^, 95% *CI* [0.36, 0.53], η_*p*_^2^ = 0.47] and *D*-value and Arrangement [*F*(4.71,339.19) = 5.37, *p* < 0.001^∗∗^, 95% *CI* [0.02, 0.12], η_*p*_^2^ = 0.07]. All three clusters of engagement ratings increase with *D*-value, but with different rates of incline.

##### Dislike-like

A 2-way 8 × 2 repeated-measures ANOVA [*D*-value (1.1, 1.2, 1.3, 1.4, 1.5, 1.6, 1.7, and 1.8) × Arrangement (randomized vs. non-randomized)] was completed to examine the impact of *D-*value and Arrangement on pattern preference (Figure 9C). A violation of the assumption of sphericity was indicated by Mauchly’s test for *D*-value [χ^2^(27) = 534, *p* < 0.001^∗∗^] and the interaction between *D*-value and Arrangement [χ^2^(27) = 108.05, *p* < 0.001^∗∗^], thus degrees of freedom were corrected using Greenhouse-Geisser estimates of sphericity (ε = 0.232 and 0.608, respectively). A significant main effect of *D*-value [*F*(1.63,120.26) = 6.71, *p* = 0.003^∗^, 95% *CI* [0.01, 0.18], η_*p*_^2^ = 0.08], Arrangement [*F*(1,74) = 19.74, *p* < 0.001^∗∗^, 95% *CI* [0.07, 0.36], η_*p*_^2^ = 0.21], and the interaction between *D*-value and pattern arrangement [*F*(4.26,314.93) = 6.05, *p* < 0.001^∗∗^, 95% *CI* [0.02, 0.13], η_*p*_^2^ = 0.08] were identified. Collapsed over pattern arrangement, average ratings of preference ranged from a low of 3.21 (*SD* = 1.6) for *D* = 1.1 to a high of 4.0 (*SD* = 1.45) for *D* = 1.6, indicating that participants’ preference for global forest fractals increases with pattern complexity. Paired samples *t*-tests revealed significant differences in preference between *D*-values (see Table 2). Comparing non-random and random arrangements, significant differences exist for *D* = 1.2 [*t*(74) = 2.66, *p* = 0.01^∗^, 95% *CI* [0.07, 0.47], *d* = 0.17], *D* = 1.5 [*t*(74) = 4.25, *p* < 0.001^∗∗^, 95% *CI* [0.26, 0.72], *d* = 0.4], *D* = 1.6 [*t*(74) = 3.93, *p* < 0.001^∗∗^, 95% *CI* [0.41, 1.26], *d* = 0.55], *D* = 1.7 [*t*(74) = 2.38, *p* = 0.02^∗^, 95% *CI* [0.07, 0.75], *d* = 0.25], and *D* = 1.8 [*t*(74) = 4.14, *p* < 0.001^∗∗^, 95% *CI* [0.43, 1.21], *d* = 0.47]. The interaction between *D*-value and Arrangement indicates that the ratings differed across arrangement type depending on *D*-value, with generally increasingly higher ratings for non-randomized compared to randomized fractal patterns as *D*-values increased.

Two subgroups were identified in ratings of pattern liking with the two step cluster analysis (Figure 9G). Mauchly’s test indicated a violation of the assumptions of sphericity for *D*-value [χ^2^(27) = 397.62, *p* < 0.001^∗∗^] and interaction between *D*-value and Arrangement [χ^2^(27) = 109.84, *p* < 0.001^∗∗^]. Therefore, degrees of freedom were corrected using Greenhouse-Geisser estimates of sphericity (ε = 0.286 and 0.603, respectively). A significant main effect of *D*-value [*F*(2.0,146.05) = 14.31, *p* < 0.001^∗∗^, 95% CI [0.06, 0.26], η_*p*_^2^ = 0.16] and Arrangement [*F*(1,73) = 19.12, *p* < 0.001^∗∗^, 95% CI [0.06, 0.36], η_*p*_^2^ = 0.21], as well as interactions between *D*-value and Clusters [*F*(2.0,146.05) = 28.72, *p* < 0.001^∗∗^, 95% CI [0.16, 0.38], η_*p*_^2^ = 0.29] as well as Arrangement and Clusters were identified [*F*(4.22,308.32) = 6.33, *p* < 0.001^∗∗^, 95% CI [0.02, 0.18], η_*p*_^2^ = 0.08]. Cluster 1, comprising 57% of the sample, shows similar ratings of pattern liking for low and moderate patterns then decreases with higher *D*-values. However, Cluster 2, comprising 43% of participants, demonstrates an increasing liking of the patterns with increasing *D*-value.

##### Tired-refreshing

A 2-way 8 × 2 repeated-measures ANOVA [*D*-value (1.1, 1.2, 1.3, 1.4, 1.5, 1.6, 1.7, and 1.8) × Arrangement (randomized vs non-randomized)] was completed to examine the impact of *D-*value and Arrangement on perceived pattern refreshment (Figure 9D). A violation of the assumption of sphericity was indicated by Mauchly’s test for *D*-value [χ^2^(27) = 853.96, *p* < 0.001^∗∗^] and the interaction between *D*-value and Arrangement [χ^2^(27) = 46.53, *p* = 0.01^∗^], thus degrees of freedom were corrected using Greenhouse-Geisser estimates of sphericity (ε = 0.174 and 0.847, respectively). The only significant effect was for Arrangement [*F*(1,74) = 15.97, *p* < 0.001^∗∗^, 95% *CI* [0.05, 0.32], η_*p*_^2^ = 0.18]. Ratings for non-randomized patterns (*M* = 3.68, *SD* = 1.53) were slightly higher than randomized patterns (*M* = 3.53, *SD* = 1.57). No significant main effect of *D*-value [*F*(1.22,90.04) = 0.14, *p* = 0.75, 95% *CI* [0, 0.05], η_*p*_^2^ = 0.002], or interaction between *D*-value and pattern arrangement [*F*(5.93,438.61) = 1.54, *p* = 0.16, 95% *CI* [0, 0.04], η_*p*_^2^ = 0.02] were found. Between non-random and random arrangements significant differences exist for *D* = 1.2 [*t*(74) = 2.22, *p* = 0.03^∗^, 95% *CI* [0.02, 0.36], *d* = 0.1], *D* = 1.6 [*t*(74) = 2.86, *p* = 0.01^∗^, 95% *CI* [0.09, 0.52], *d* = 0.27] and *D* = 1.7 [*t*(74) = 2.63, *p* = 01^∗^, 95% *CI* [0.07, 0.47], *d* = 0.18]. No subgroups were found amongst participant ratings of fractal pattern refreshment.

##### Relaxing-tense

A 2-way 8 × 2 repeated-measures ANOVA [*D*-value (1.1, 1.2, 1.3, 1.4, 1.5, 1.6, 1.7, and 1.8) × Arrangement (randomized vs. non-randomized)] was completed to examine the impact of *D-*value and Arrangement on perceptions of patterns tension (non-relaxing quality) (Figure 9E). A violation of the assumption of sphericity was indicated by Mauchly’s test for *D*-value [χ^2^(27) = 560.25, *p* < 0.001^∗∗^] and interaction between *D*-value and Arrangement [χ^2^(27) = 52.99, *p* = 0.002^∗^], thus degrees of freedom were corrected using Greenhouse-Geisser estimates of sphericity (ε = 0.218 and 0.842, respectively). A sole significant main effect of *D*-value was identified [*F*(1.53,113.17) = 140.86, *p* < 0.001^∗∗^, 95% *CI* [0.05, 0.32], η_*p*_^2^ = 0.66]. Thus no main effect of Arrangement [*F*(1,74) = 2.01, *p* = 0.16, 95% *CI* [0, 0.05], η_*p*_^2^ = 0.03] or significant interaction between *D*-value and pattern Arrangement were found [*F*(5.89,436.1) = 1.38, *p* = 0.22, 95% *CI* [0, 0.04], η_*p*_^2^ = 0.02]. Average ratings of pattern relaxation ranged from a low of 2.28 (*SD* = 1.29) for *D* = 1.1 to a high of 5.63 (*SD* = 1.28) for *D* = 1.8, suggesting that participants perceived patterns as more tense with increasing *D-*value. Paired samples *t*-tests revealed significant differences in perceived relaxation for all pairs of *D*-values (Table 2).

Three subgroups of participant perceptions of tension/relaxation were identified through two step cluster analysis (Figure 9H). Mauchly’s test indicated a violation of the assumptions of sphericity for *D*-value [χ^2^(27) = 259.97, *p* < 0.001^∗∗^] and interaction between *D*-value and Arrangement [χ^2^(27) = 52.56 *p* = 0.002^∗^]. Therefore, degrees of freedom were corrected using Greenhouse-Geisser estimates of sphericity (ε = 0.367 and 0.839, respectively). A significant main effect of *D*-value [*F*(2.57,185.17) = 101.1 *p* < 0.001^∗∗^, 95% *CI* [0.49, 0.65], η_*p*_^2^ = 0.58] and interaction between *D*-value and Clusters [*F*(5.14,185.17) = 41.63, *p* < 0.001^∗∗^, 95% *CI* [0.42, 0.6], η_*p*_^2^ = 0.54] emerged in the analysis. Cluster 1 containing 64% of participants as well as cluster 2 containing 19% of participants both produced a perceptual trend in which ratings of tension increased with pattern complexity. Cluster 3 containing the remaining 17% of the sample, produces a flat trend in ratings of pattern tension/relaxation.

Overall, we find that bipolar ratings of fractal ‘global-forest’ pattern complexity, preference, and engagement increase with additional *D*-value, whereas ratings of relaxation decrease with additional *D*-value. Perceptions of pattern refreshment are impacted by participant membership to contradictory rating trends, producing greater variance in ratings thus a flatter trend in relaxation ratings.

#### Discussion

Experiment 1B expands our investigation of psychological effects of these installed patterns but incorporates a different population of viewers and bipolar rating design. In this iteration of the perceptual rating task, participants are still recruited from a college population but from a different continent in the opposing global hemisphere (Australia) with a very different natural landscape. The rating task is also altered such that participants are instructed to rate their perception of images on a larger sliding scale between two opposing descriptors. Even with a new population and expanded study design results are highly similar to Experiment 1A. Similar to Experiment 1A, complexity, engaging, and preference ratings of ‘global-forest’ patterns all increase with increasing *D*-value; perceptions of pattern relaxation (taken as the reversed rating of ‘tense’ for this experiment) decrease with *D*-value; and refreshment did not change with *D*-value.

## Experiment 2 – Perception of Fractal “Tree-Seed” Patterns

### Materials and Methods

#### Stimuli

Experiment 2 isolates the local components of the ‘global-forest’ patterns. These local ‘tree-seed’ patterns represent a local fractal pattern composed of rectangular ‘seeds’ with locations determined by the generated flightpath (see the description of the generation method in the Introduction and Experiment 1A). The stimuli consisted of a total of 20 patterns, with 5 examples each of 4 *D*-values (*D* = 1.2, 1.4, 1.6, and 1.8). Figure 5E shows an example pattern from each *D*-value.

#### Participants

To identify the locus of these perceptual trends, 39 participants comprised of undergraduate Psychology students from the University of Oregon were recruited for the current study through the SONA participant pool system (22 females, age ranging between 18 and 29 years old, mean age 20 years old). Informed consent was acquired following a protocol approved by the Institutional Review Board at the University of Oregon and all participants received class credit for their participation.

#### Visual Displays

Experiment 2 was programmed in PsychoPy3 and presented using the online research study platform of Pavlovia (Peirce et al., 2019). This study was completed on participants’ personal computers with program stimuli scaled to the individual computer’s respective full-screen dimensions.

#### Design and Procedure

Participants viewed a series of fractal “tree-seed” patterns presented in five randomized blocks. Each block’s stimulus set consisted of 5 unique patterns ranging across 4 levels of complexity or *D*-value (*D* = 1.2, 1.4, 1.6, and 1.8). A slider response task was used to self-report ratings for each fractal pattern, resulting in 20 trials per block. Each block consisted of a singular judgment type (complexity, engaging, preference, refreshing, or relaxing).

Before each block, participants were instructed to make a single randomly ordered judgment (complexity, preference, engaging, refreshing, or relaxing) for each stimulus presented in that block. Specifically, they were asked to answer one of 5 questions for each block: “How _______ is the image?” with one of 5 different words placed in the blank (complex, engaging, preferable, refreshing, relaxing). They were told to indicate their rating of each given pattern on a slider ranging between 0 and 1 located below the image, with the “0” end of the slider indicating “not at all” and the “1” end of the slider indicating “completely.” They were asked to use the full range of the slider and to click on the slider to indicate their rating. Periodically, an attention check trial appeared in which participants were instructed to select either “0” or “1.” The images remained on the screen until participants selected their rating. Upon completion of the experiment, participants completed a demographic questionnaire and were debriefed according to the protocols approved by the Institutional Review Board at the University of Oregon.

### Results

Data from 39 adult participants (between 18 and 29 years old) were retained from the 60 adults who participated in the experiment. Data were excluded due to: (a) failure to complete the study, (b) failure of greater than 3 attention checks, or (c) recording the same rating for greater than four consecutive trials.

#### Fractal Judgment Task

**FIGURE 10:**
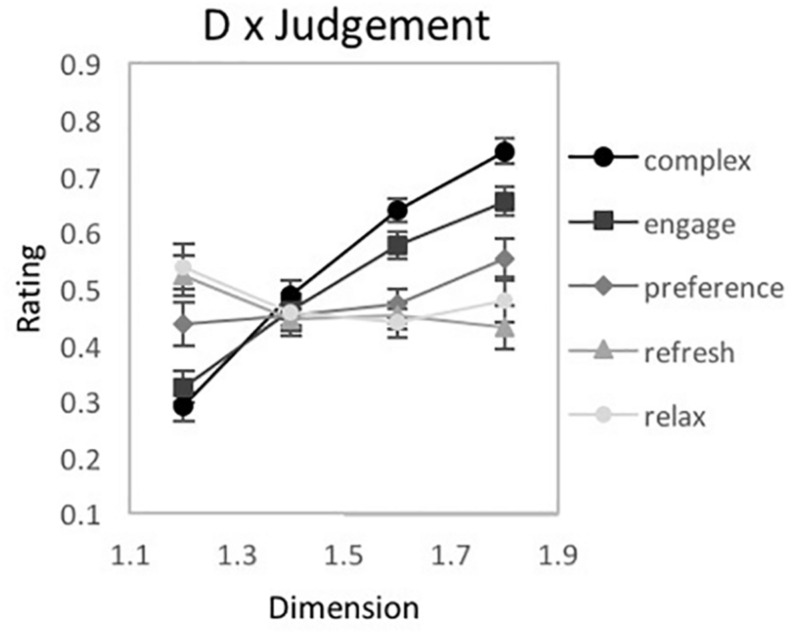
Experiment 2 results for ‘tree-seed’ fractal patterns using a unipolar rating scale. Results show a significant interaction between fractal dimension (*D*) and judgment type (complex, engaging, preferred, refreshing, and relaxing). Participant rating (on a scale from 0 to 1) is plotted as a function of *D*-value for the different judgment conditions.

A 2-way repeated-measures 4 × 5 ANOVA [*D*-value (1.2, 1.4, 1.6, and 1.8) × Judgment (complexity, engaging, preference, refreshing, relaxing)] was performed using IBM SPSS Statistics for Macintosh (Version 25.0) on rating data for the fractal patterns (recorded as location selected on a rating response slider). Mauchly’s test indicated a violation of the assumption of sphericity for *D*-value [χ^2^(5) = 59.58, *p* < 0.001^∗∗^], Judgment [χ^2^(9) = 25.24, *p* = 0.003^∗^], and the interaction of *D*-value and Judgment [χ^2^(77) = 216.75, *p* < 0.001^∗∗^]. Therefore, degrees of freedom were corrected using Greenhouse-Geisser estimates of sphericity (ε = 0.525, 0.732, and 0.463, respectively). Indicated by a double asterisk for significance of *p* < 0.001 and single asterisk for significance of *p* < 0.05, significant main effects of *D*-value [*F*(1.58, 59.88) = 12.64, *p* < 0.001^∗∗^, 95% *CI* [0.07, 0.4], η_*p*_^2^ = 0.25] and Judgment [*F*(2.93, 111.29) = 5.55, *p* = 0.002^∗^, 95% *CI* [0.02, 0.23], η_*p*_^2^ = 0.13], as well as an interaction between *D*-value and Judgment emerged [*F*(5.56, 211.26) = 17.88, *p* < 0.001^∗∗^, 95% *CI* [0.21, 0.4], η_*p*_^2^ = 0.32]. For the *D*-value and Judgment interaction, some judgments had ratings that increased in value with *D* (complexity, engagement, preference), while others were relatively flat or slightly decreasing (refreshing, relaxing) (Figure 10). Similar to the prior experiments, a series of planned comparisons explored the locus of the significant interaction between *D*-value and Judgment using ANOVAs, paired *t*-tests (Table 3), as well as a 2-step clustering analysis to determine if subgroups could further explain perceptual trends.

**TABLE 3:** Experiment 2-paired samples *t*-tests across *D*-value and judgment.

	Complex	Engaging	Preference	Refreshing	Relaxing
D = 1.2 vs. D = 1.4	t = −10.01** (d = 1.29)	t = −4.49** (d = 0.8)	t = −0.36 (d = 0.05)	t = 2.11* (d = 0.33)	t = 2.13* (d = 0.38)
D = 1.2 vs. D = 1.6	t = −11.69** (d = 2.35)	t = −6.02** (d = 1.61)	t = −0.71 (d = 0.15)	t = 1.42 (d = 0.37)	t = 1.98 (d = 0.53)
D = 1.2 vs. D = 1.8	t = −13.39** (d = 3.21)	t = −7.33** (d = 1.94)	t = −1.77 (d = 0.49)	t = 1.36 (d = 0.39)	t = 0.90 (d = 0.29)
D = 1.4 vs. D = 1.6	t = −6.30** (d = 1.03)	t = −3.67** (d = 0.77)	t = −0.71 (d = 0.12)	t = −0.20 (d = 0.0)	t = 0.66 (d = 0.13)
D = 1.4 vs. D = 1.8	t = −8.41** (d = 1.71)	t = −5.38** (d = 1.15)	t = −2.17* (d = 0.51)	t = 0.27 (d = 0.09)	t = −0.42 (d = 0.05)
D = 1.6 vs. D = 1.8	t = −5.22** (d = 0.77)	t = −3.45** (d = 0.47)	t = −2.47* (d = 0.44)	t = 0.60 (d = 0.10)	t = −1.37 (d = 0.15)

*Indicates significance of p < 0.05. **Indicates significance of p < 0.001.

##### Complexity

**FIGURE 11:**
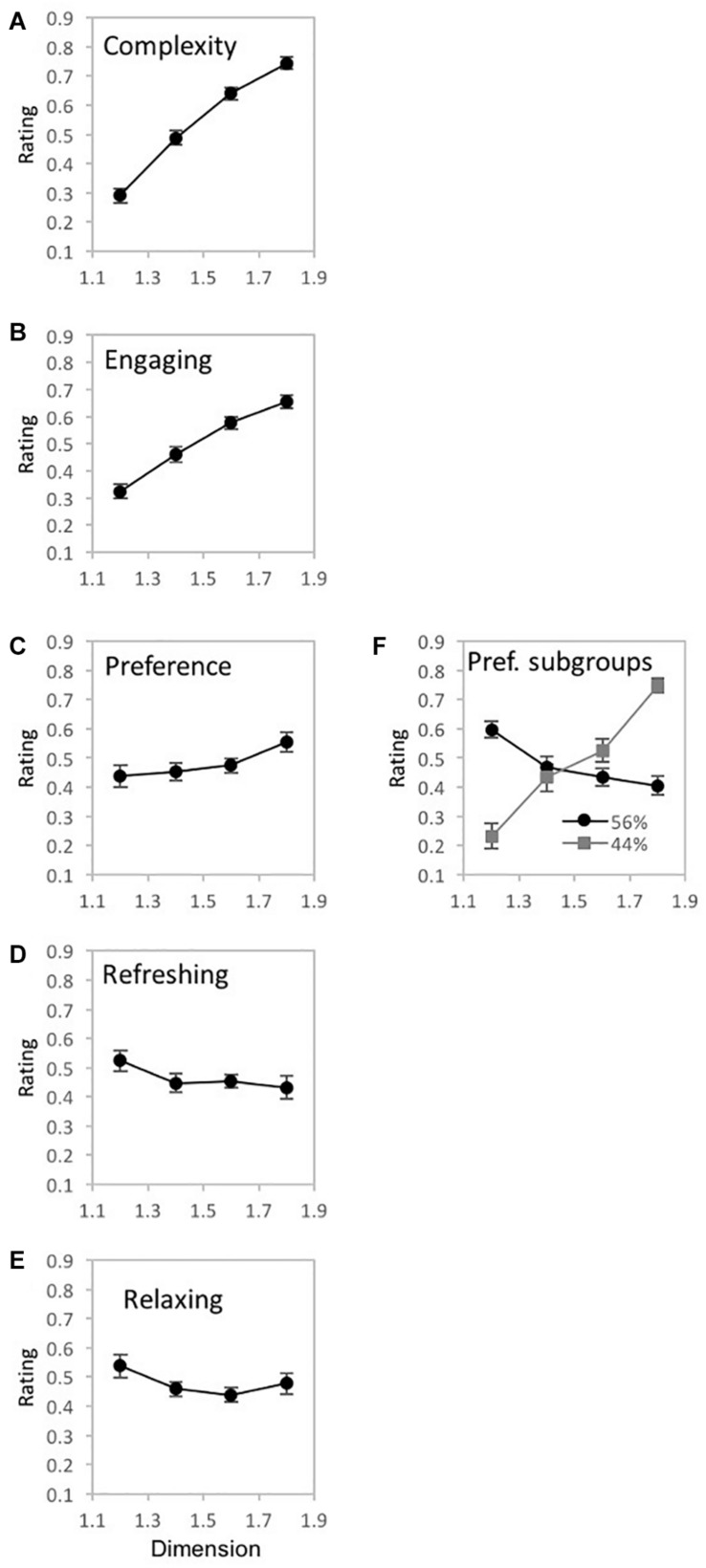
Experiment 2 results for ‘tree-seed’ fractal patterns for 5 different judgment conditions (how complex, engaging, preferred, refreshing, and relaxing). **(A–E)** shows plots of mean ratings as a function of fractal dimension (*D*) for the different judgment conditions (error bars represent standard error). **(F)** shows a plot of the mean ratings as a function of fractal dimension (*D*) for each subpopulation identified with cluster analysis (error bars represent standard error).

A one-way repeated measures ANOVA was completed on the effects of *D*-value on ratings of pattern complexity (Figure 11A). Mauchly’s test indicated a violation of the assumption of sphericity for *D*-value [χ^2^(5) = 28.03, *p* < 0.001^∗∗^], thus, degrees of freedom were corrected using Greenhouse-Geisser estimates of sphericity (ε = 0.659). A significant main effect of *D*-value [*F*(1.98, 75.08) = 107.58, *p* < 0.001^∗∗^, 95% *CI* [0.63, 0.8], η_*p*_^2^ = 0.74] was detected. Average complexity ratings ranged from a low of 0.29 (*SD* = 0.15) for *D* = 1.2 to a high of 0.74 (*SD* = 0.13) for *D* = 1.8, indicating that participants perceive greater complexity with the presence of higher *D*-value. Paired samples *t*-tests revealed significant differences in perceived complexity between all pairs of *D*-values (Table 3). A 2-step clustering analysis identified no significant subgroups for pattern complexity.

##### Engaging

A one-way repeated measures ANOVA was completed on the effects of *D*-value on ratings of pattern engagement (Figure 11B). Mauchly’s test indicated a violation of the assumption of sphericity for *D*-value [χ^2^(5) = 28.01, *p* < 0.001^∗∗^], thus, degrees of freedom were corrected using Greenhouse-Geisser estimates of sphericity (ε = 0.660). A significant main effect of *D*-value exists for ratings of pattern engagement [*F*(1.98, 75.24) = 33.07, *p* < 0.001^∗∗^, 95% *CI* [0.29, 0.58], η_*p*_^2^ = 0.47]. Average engagement ratings ranged from a low of 0.32 (*SD* = 0.18) for *D* = 1.2 to a high of 0.65 (*SD* = 0.16) for *D* = 1.8, suggesting that patterns are perceived as more engaging with the introduction of higher *D*-values. Paired samples *t*-tests revealed significant differences in perceived engagement between all pairs of *D*-values (Table 3). No clusters were found amongst the participant ratings of pattern engagement.

##### Preference

A one-way repeated measures ANOVA was completed on the effects of *D*-value on ratings of pattern preference (Figure 11C). Mauchly’s test indicated a violation of the assumption of sphericity for *D*-value [χ^2^(5) = 42.16, *p* < 0.001^∗∗^]. Therefore, degrees of freedom were corrected using Greenhouse-Geisser estimates of sphericity (ε = 0.586). No significant main effect of *D*-value was identified [*F*(1.58, 66.76) = 2.54, *p* = 0.09, 95% CI [0, 0.18], η_*p*_^2^ = 0.06]. Paired samples *t*-tests did reveal significant differences in preference between *D*-values (see Table 3) indicating a trend of higher preference ratings for patterns with higher *D*-values, at least among *D*-values of 1.4, 1.6, and 1.8.

Two subgroups emerged in the 2-step cluster analysis (Figure 11F). Mauchly’s test indicated a violation of the assumptions of sphericity for *D*-value [χ^2^(5) = 11.89, *p* = 0.04^∗^]. Therefore, degrees of freedom were corrected using Greenhouse-Geisser estimates of sphericity (ε = 0.861). Both a significant main effect of *D*-value [*F*(2.58, 95.58) = 9.29, *p* < 0.001^∗∗^, 95% *CI* [0.06, 0.32], η_*p*_^2^ = 0.2], and significant interaction between *D*-value and sub-groups [*F*(2.58, 95.58) = 42.07, *p* < 0.001^∗∗^, 95% *CI* [0.38, 0.62], η_*p*_^2^ = 0.53] emerged. Cluster 1 comprised 56% of the sample and represents a trend of fractal preference peaking at the lowest *D*-value and decreasing with added complexity. Conversely, cluster 2 which accounts for the remaining 44% of the sample, represents an opposing trend with fractal preference increasing steeply with *D*-value and peaking at the highest complexity.

##### Refreshing

A one-way repeated measures ANOVA was completed on the effects of *D*-value on ratings of pattern refreshment (Figure 11D). Mauchly’s test indicated a violation of the assumption of sphericity for *D*-value [χ^2^(5) = 54.02, *p* < 0.001^∗∗^]. Thus, degrees of freedom were corrected using Greenhouse-Geisser estimates of sphericity (ε = 0.554). No significant main effect of *D*-value was identified in the data [*F*(1.66, 63.11) = 1.49, *p* = 0.24, 95% *CI* [0, 0.15], η_*p*_^2^ = 0.04]. Paired samples *t*-tests revealed one significant difference in perceived refreshment between *D* = 1.2 and *D* = 1.4 (see Table 3). No subgroup clusters were identified in the data.

##### Relaxing

A one-way repeated measures ANOVA was completed on the effects of *D*-value on ratings of pattern relaxation (Figure 11E). Mauchly’s test indicated a violation of the assumption of sphericity for *D*-value [χ^2^(5) = 64.07, *p* < 0.001^∗∗^]. Therefore, degrees of freedom were corrected using Greenhouse-Geisser estimates of sphericity (ε = 0.489). No significant main effect of *D*-value was identified in the data [*F*(1.47, 55.72) = 1.82, *p* = 0.18, 95% *CI* [0, 0.18], η_*p*_^2^ = 0.05]. Paired samples *t*-tests revealed a sole significant difference in perceived relaxation between *D* = 1.2 and *D* = 1.4 (see Table 3). No additional clusters are found amongst the participant ratings of pattern relaxation.

### Discussion

Experiment 2 maintains the same methodological structure and perceptual decisions as Experiment 1A but replaces the ‘global-forest’ pattern with fractal ‘tree-seed’ patterns. Similar to Experiment 1, judgments of complexity and engagement increase with *D*-value and there is a trend for higher preference ratings for patterns with higher *D*-values and 2 subgroups with opposing responses for preference for pattern complexity. The smaller sample size in Experiment 2 may have affected the strength of the overall positive trend. Also similar to Experiment 1, judgments for refreshing are similar across *D*-value. However, unlike Experiment 1, judgments of relaxing remain the same, rather than decrease, with *D*-value.

## General Discussion

Evaluations of Euclidean human-made space can be altered by integrating the aesthetics of nature (Taylor et al., 2005; Hagerhall et al., 2015). Increased time spent amongst unnatural Euclidean structures is associated with higher rates of visual strain, headaches, and overall stress resulting from additional effort exerted by the visual system to process more artificial patterns (Hagerhall et al., 2008; O’Hare and Hibbard, 2011; Penacchio and Wilkins, 2015; Le et al., 2017; Ogawa and Motoyoshi, 2020). Fractal patterns have the opportunity to combat these negative effects of unnatural environments by introducing easy to visually process natural patterns that can alter the occupants’ experience of a space. Previous research has shown that preference for statistically generated fractal patterns peaks at low-moderate fractal dimension (“*D*-value”), a level of complexity that is prevalent in nature. In contrast, preference for exact fractals peaks at higher *D*-values due to the increased order introduced by their exact repetition (Bies et al., 2016). In order to maximize the possible positive effects of a composite fractal design that may provide greater flexibility to be used in installations that vary in media, location, and artistic style, the current set of studies explores a novel range of perceptual responses to fractal designs that expands beyond typical measurements of viewer preference in order to categorize trends in fractal perception for individual and group profiles taken from a more expansive sample of observers.

Across 3 experiments that vary in stimulus pattern composition, participant population, and rating scale we find similar trends in fractal perception. Experiment 1 used ‘global-forest’ fractal designs to demonstrate that ratings of pattern complexity, engagement, and preference increase with fractal complexity or *D*-value. In contrast, perception of pattern refreshment stays constant across *D*-value while perception of relaxation decreases with increasing *D*-value. Experiment 2 investigates the contribution of the local ‘tree-seed’ patterns to ratings of the “global-forest” designs. By replicating Experiment 1A using images of individual ‘tree-seed’ patterns that feature in the global design, we are able to get a measure of the contribution of the local patterns to the ratings of the overall composite design. Results demonstrate that most of the trends in participant ratings remain consistent with those of the overall fractal installation design. Specifically, perceptions of pattern complexity and engagement, and to a certain extent preference, all increase with increasing *D*-value. Also similar are judgments for refreshing which in all experiments are similar across *D*-value. However, unlike Experiment 1, judgments of relaxing remain the same, rather than decrease, with *D*-value. These results suggest that the local patterns contribute to the perception of the global design. Thus, across this set of studies, robust perceptions of fractal patterns remain consistent across countries, methodology, and to a certain extent, pattern design. The use of unipolar and bipolar scales between Experiments 1A and 1B show similar overall trends for the ‘global-forest’ fractal designs.

Across both studies subgroupings have a significant impact on overall trends, supporting previous findings of individual differences in preference for fractal complexity (Bies et al., 2016; Spehar et al., 2016; Street et al., 2016). Opposing subgroup trends are found for perceptual ratings of preference, refreshing, and relaxing. The opposing nature of these subgroups can serve to inform industrial design choices when selecting fractal patterns for installation by taking into consideration the *D*-value with the greatest agreement amongst individuals for the various judgments, thus benefiting the majority of occupants without negatively affecting the experience of subgroups of occupants. Specifically, if the goal is to optimize the engagement, preference, refreshment, and relaxation qualities of the fractal design across participants, then a pattern with mid-high *D*-value would provide this optimal balance, since these patterns have the greatest agreement among individual participant ratings for preference, engagement and refreshment while maintaining mid-range relaxing effects that become much lower for patterns with the highest *D*-values. More generally, our results highlight the potential of fractals for human-centered design - the choice of *D* value might ultimately depend on both the occupants and the functionality of the space (e.g., classrooms might be different from hospitals).

Both studies also demonstrate an effect of pattern randomization, whereby ratings of engagement, preference, refreshing, and relaxing qualities are slightly higher for non-randomized compared to randomized patterns. Fortunately, for many of the installations (e.g., carpets and projected light patterns) the visibility of the edges defining the randomly positioned tiles is much less apparent in the installations than in the randomized patterns presented here.

Lastly, the similarity between findings of Study 1A and 1B are not impacted by the geographical location of participants. Our findings suggest that perceptions of fractal patterns are not altered by the diverse natural environments where participants reside. This result supports the finding that preference for fractal complexity forms early in human development (sometime prior to three years of age) and is not further altered by life experience in western participants (Robles et al., 2020). Although this study recruits from a broader group of participants, our findings are still limited due to the overarching homogeneity in “WEIRD” participant samples. However, the addition of variation in geographical location and composition of cultural subgroupings suggests that these consistent perceptions of fractal patterns are experienced by broader populations around the global, thus encouraging further studies addressing fractal perceptions in more diverse samples. Taken as a whole, findings from Experiment 1 lends support to possible universality of fractal pattern perception, despite variability in testing methods, individual differences driving rating subgroups, and samples coming from experience with two different natural landscapes.

The “global-forest” patterns with *D* = 1.6 have already been installed into humanmade spaces in hopes of reducing occupant stress while increasing the aesthetic experience of the space. For patterns to successfully decrease stress levels, they must elicit lower physiological arousal and provide a restorative effect for attention (Hagerhall et al., 2015). Relaxation and refreshment coincide with lower levels of arousal whereas engagement requires elevated levels of arousal. For installations to be effective without altering the overall aesthetics of the space, patterns must balance desirable levels of preference and engagement with relaxing and refreshing qualities. Unlike the fractal preference for natural statistical fractals (Spehar et al., 2016), preference for the current fractal patterns increase with increasing *D*-value more similar to fractal patterns that repeat in an exact manner (Bies et al., 2016). This result may be due to a number of factors including: (1) an increased preference for patterns with higher element density that is present in the higher *D*-value patterns; (2) the introduction of Euclidean structure and exact repetition found in the repeating rows of the ‘tree-seed’ patterns; and (3) the visibility of the square-shaped seed pattern that is used to grow the fractal ‘tree-seed’ patterns. For a biophilic installation to have the greatest stress reducing effect, the fractal design would be required to possess a mid-high *D*-value which would maintain elevated pattern preference, but not suffer from the steepest decline in pattern relaxation that occurs at the highest *D*-values. The fractal patterns employed in the installations shown in Figure 4 all have these optimal mid-high *D*-values.

Future studies will further explore the ways in which these fractal designs impact occupants’ perceptions by expanding our studies to assess the extent to which our findings apply to broader populations of participants, additional changes in pattern design (including different local components, global flight-path arrangements, and global design), and can be directly identified with changes in physiological and verbal measures of stress and arousal. Further replications will be conducted utilizing Virtual Reality (VR) to assess responses to these patterns installed in 3-dimensional architectural spaces in order to more directly manipulate participant experience and measure changes in psychological effects in an immersive environment. By balancing perceptual factors, patterns can be produced and installed to maximize aesthetic experiences of particular spaces. The collaboration of design, physics, psychology, and technology provides a vital opportunity to test for and determine visual patterns that produce optimal perceptual responses and experiences in occupants of human-made structures. By selecting fractal patterns with *D*-values that are appropriate for particular built environments and mediums, instillations of these natural patterns have the opportunity to decrease eye-strain, headache rates, and stress (O’Hare and Hibbard, 2011; Penacchio and Wilkins, 2015; Le et al., 2017) in a large percentage of viewers (Bies et al., 2016; Street et al., 2016; Pyankova et al., 2019) while potentially increasing the aesthetic experience of the space.

## Data Availability Statement

The datasets presented in this study can be found in online repositories. The names of the repository/repositories and accession number(s) can be found below: https://doi.org/10.7910/DVN/VZE4G4. Program codes are available upon request.

## Ethics Statement

The studies involving human participants were reviewed and approved by Institutional Review Board at the University of Oregon and UNSW Human Research Advisory Panel. The patients/participants provided their written informed consent to participate in this study.

## Author Contributions

KR, RT, BS, and MS contributed to the study design. RT, JS, CR, SM, SS, AL, and ML contributed to stimulus generation. KR and CV contributed to programming the experiments. KR and BS contributed to testing and data collection. KR, MS, and BS contributed to the data analysis and interpretation. KR and MS drafted the manuscript. All authors contributed to the manuscript editing and approved the final version of the manuscript for submission.

## Conflict of Interest

SS, AL, and ML were employed by company 13&9 Design, Austria. The remaining authors declare that the research was conducted in the absence of any commercial or financial relationships that could be construed as a potential conflict of interest.

## Publisher’s Note

All claims expressed in this article are solely those of the authors and do not necessarily represent those of their affiliated organizations, or those of the publisher, the editors and the reviewers. Any product that may be evaluated in this article, or claim that may be made by its manufacturer, is not guaranteed or endorsed by the publisher.
